# Gut pathobiome mediates behavioral and developmental disorders in biotoxin-exposed amphibians

**DOI:** 10.1016/j.ese.2024.100415

**Published:** 2024-03-21

**Authors:** Qianqian Pan, Tianxing Lv, Haorong Xu, Hongda Fang, Meng Li, Jiaping Zhu, Yue Wang, Xiaoyan Fan, Ping Xu, Xiuguo Wang, Qiangwei Wang, Haruna Matsumoto, Mengcen Wang

**Affiliations:** a Ministry of Agricultural and Rural Affairs Laboratory of Molecular Biology of Crop Pathogens and Insects, Zhejiang University, Hangzhou, 310058, China; b Institute of Pesticide and Environmental Toxicology, College of Agriculture and Biotechnology, Zhejiang University, Hangzhou, 310058, China; c Institution of Tea Science, Zhejiang University, Hangzhou, 310058, China; d The Tobacco Research Institute, Chinese Academy of Agricultural Sciences, Qingdao, 266101, China; e Global Education Program for AgriScience Frontiers, Graduate School of Agriculture, Hokkaido University, Sapporo, Japan

**Keywords:** Gut microbiome, Pathobiome, Biotoxin pollution, Developmental toxicity, Behavioral disorders

## Abstract

Emerging evidence suggests a link between alterations in the gut microbiome and adverse health outcomes in the hosts exposed to environmental pollutants. Yet, the causal relationships and underlying mechanisms remain largely undefined. Here we show that exposure to biotoxins can affect gut pathobiome assembly in amphibians, which in turn triggers the toxicity of exogenous pollutants. We used *Xenopus laevis* as a model in this study. Tadpoles exposed to tropolone demonstrated notable developmental impairments and increased locomotor activity, with a reduction in total length by 4.37%–22.48% and an increase in swimming speed by 49.96%–84.83%. *Fusobacterium* and *Cetobacterium* are predominant taxa in the gut pathobiome of tropolone-exposed tadpoles. The tropolone-induced developmental and behavioral disorders in the host were mediated by assembly of the gut pathobiome, leading to transcriptome reprogramming. This study not only advances our understanding of the intricate interactions between environmental pollutants, the gut pathobiome, and host health but also emphasizes the potential of the gut pathobiome in mediating the toxicological effects of environmental contaminants.

## Highlights

• The amphibians populations in paddies decline with increasing tropolone levels.
• Tropolone exposure leads to behavioral and developmental disorders in tadpoles.
• Core gut pathobiome taxa are identified in tropolone-exposed tadpoles.
• Gut pathobiome drivers toxicity from tropolone exposure.

## Graphical abstract

**Figure:**
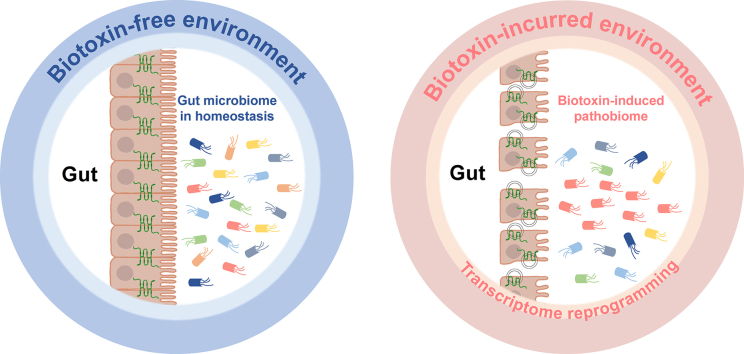

## 1 Introduction

Amphibians play an essential role in sustaining ecosystem fitness and stability [1]. However, more than 500 amphibian species are currently facing population declines and extinctions at a global scale [2,3]. These ecological consequences have been proposed to be associated with various environmental pollutants, such as agrochemicals, heavy metals, and emerging biotoxins [[4], [5], [6], [7]]. As the largest agroecosystem and wetland ecosystem on Earth, the paddy ecosystem is a dominant habitat for amphibians [8]. However, a wide array of phytopathogen-derived biotoxins, such as tropolone (TR) [9], ustiloxins [10,11], and pyricuol [12], have been frequently detected in soils and sediments, especially in aquatic compartments [13]. Biotoxin pollution has become a growing global concern due to its adverse impact on public health, but its ecological effects on amphibian populations are still underestimated.

**Fig. 1:**
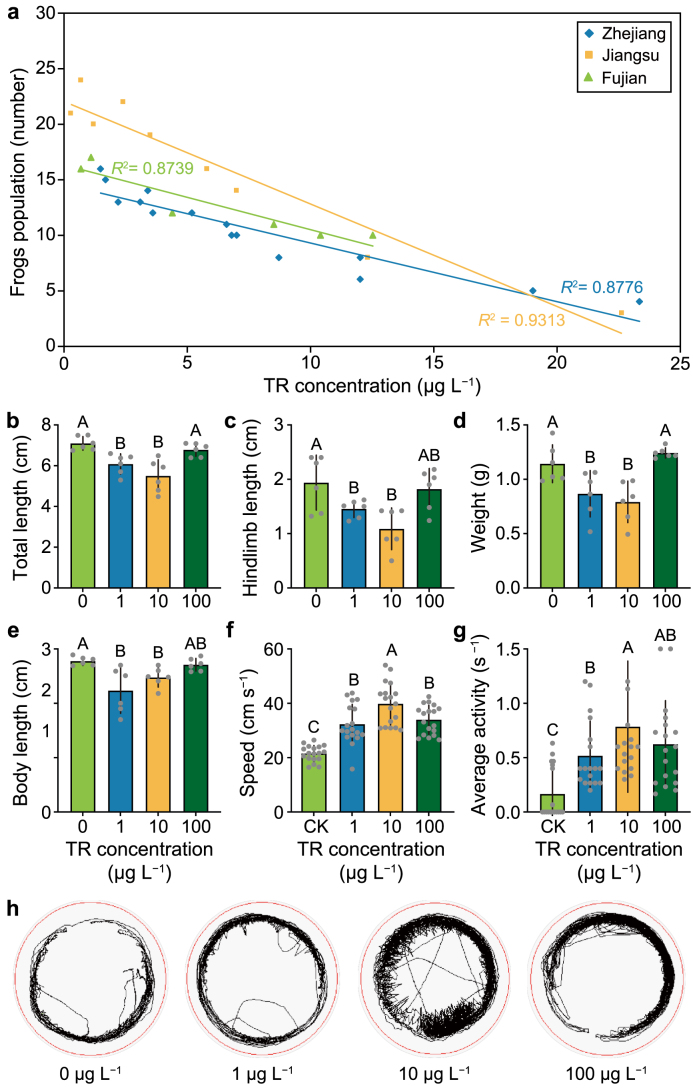
**Alterations in the density of frogs in paddy fields and development and locomotor activity of the tadpoles upon exposure to biotoxin. a**, Regression analysis of the density of the frog population and the biotoxin tropolone (TR) levels in different region-specific rice paddies (Zhejiang, Jiangsu, and Fujian). **b–e,** Effects of biotoxin tropolone (TR) exposure (TR at 0, 1, 10, and 100 μg L^−1^) on the total length (**b**), hindlimb length (**c**), weight (**d**), and body length (**e**) of tadpoles (*n* = 6 replicates). **f**–**g,** Comparison of swimming speed (**f**) and average activity (**g**) of tadpoles in the control (CK: TR at 0 μg L^−1^) and TR-exposed groups (TR at 1, 10, and 100 μg L^−1^) (*n* = 18 replicates). **h,** Visualization of the swimming speed and average activity of tadpoles in the control (TR at 0 μg L^−1^) and TR-exposed groups (TR at 1, 10, and 100 μg L^−1^). The whole tracking of tadpoles recorded for 10 min is shown. Different letters with error bars indicate a significant difference based on a one-way analysis of variance (ANOVA) with least significant difference (LSD) test (*p* < 0.05).

TR is a predominant biotoxin produced by the agricultural pathogen *Burkholderia plantarii* (*B. plantarii*). It is characterized as a seven-membered aromatic ring compound with the molecular formula C_7_H_6_O_2_ (Fig. S1), displaying high solubility in both organic solvents and water [9]. As one of the most commonly identified biotoxins in agroecosystems, TR has been consistently detected in most paddy fields, with concentrations ranging from 14 to 157 μg L^−1^, as observed in a continental-scale pilot monitoring [9]. This *B. plantarii*-derived biotoxin has been observed to exhibit varying degrees of acute toxicities toward microbes, higher plants, and mammals [[14], [15], [16]]. Furthermore, we recently observed a negative correlation between the population of frogs, the predominant amphibian species, and exposure to trace levels of TR (ranging from 0.3 to 23.3 μg L^−1^) in the paddy aquatic environment. (Fig. 1a, Fig. S2, Dataset 1). This suggests that exposure to this prevalent biotoxin during the developmental stages of frogs, particularly the sensitive early developmental stages, could adversely affect the overall population stability of frogs within paddy ecosystems. While tadpoles are commonly recognized as the most vulnerable early developmental stage to environmental contaminants throughout a frog's lifespan, the specific impacts of TR under environmentally relevant exposures remain largely unknown.

Like most animals, gut microbiome homeostasis plays a vital role in maintaining the development and health of amphibians [[17], [18], [19]]. Antibiotics, drugs, and pesticides are examples of external stimuli that have been shown to negatively affect the homeostasis of the gut microbiome, resulting in a shift from a symbiotic microbiome to a dysbiotic microbiome [20]. A dysbiotic microbiome is generally associated with a decreasing (or potentially decreasing) health status of hosts and with the onset of diseases and pathological intestinal microenvironments associated with obesity, malnutrition, and chronic inflammatory diseases [[21], [22], [23], [24]]. Recently, a dysbiotic gut microbiome resulting from the disruption of a symbiotic gut microbiome that promotes health and is environmentally stable has been conceptualized as the pathobiome [25,26]. Recent research has shown that the gut pathobiome influences immune modulation in sepsis patients following surgical injury [27]. Preliminary data from several perspectives suggest a direct link between the gut pathobiome and a maladaptive immune response in incritical illness [28]. Apart from the characterized associative relationship between the pathobiome and disrupted host health, little is known about the causal relationship between them, particularly upon exposure to extrinsic cues at the environmental levels.

In this study, we utilized the amphibian frog model organism *Xenopus laevis* (*X. laevis*) to investigate how exposure to trace levels of TR influences host development and behavior, specifically focusing on gut microbiome alterations. Exposure to TR impaired development and increased locomotor activity in *X. laevis*. Further, through integrating high-throughput data, behavioral and transcriptomic analyses, and microbiome transplantation experiments, we uncovered the core taxa within the pathobiome and elucidated the mechanism underlying the pathobiome-mediated host dysfunction. Our study unravels the previously hidden role of the gut pathobiome in the ecotoxicity caused by environmental pollutants to the host.

## 2 Materials and methods

### 2.1 Chemicals and reagents

Tropolone (TR), 2-hydroxy-2,4,6-cycloheptatrien-1-one, with a purity of 98.0%, was obtained from TCI. Ethylacetate (EtOAc) and other organic solvents (chromatographic grade) were purchased from Merck. Agar powder was purchased from Wako. Tricaine mesylate (MS-222) and dimethyl sulfoxide (DMSO) were purchased from Sigma (St. Louis, USA). The DisQue kit, purchased from Waters, was used to extract and purify TR.

### 2.2 Investigation of the amphibian population and quantification of TR *in situ*

To investigate the relationship between TR concentrations and amphibian population, we conducted a pilot study in Zhejiang, Jiangsu, and Fujian Provinces in China. The population of frogs was calculated in 100 plants per paddy field via the chessboard-type sampling method. The paddy water (2 L) was collected at each point by using a five-point sampling method and subjected to determination of TR after filtration through No. 101 filter paper (Advantec) in a Buchner's funnel [9,29,30] (Fig. S3). The extraction and purification of TR were performed according to previous methods [8], and further TR was subjected to analysis as follows. The filtered paddy water (approximately 50 mL) was transferred into a 250 mL separatory funnel. After 1 min of homogenization, an aliquot of the upper layer (25 mL) was pipetted into a 50 mL DisQuE centrifuge tube preloaded with 6 g of MgSO_4_ and 1.5 g of CH_3_COONa. The mixture was then centrifuged again at 4000 rpm for 1 min, vigorously shaken for 1 min, and subsequently centrifuged at 4000 rpm for 1 min. For further purification, the supernatant was concentrated by a rotatory evaporator at 45 °C. The residue was redissolved in 2 mL EtOAc and filtered through a 0.22 μM filter for quantitative analysis of TR by gas chromatography (GC)-triple quadrupole mass spectrometry (QqQ, MS/MS) (Agilent 7000C, USA). The qualification and quantification of TR were referred to the previously established methods in the laboratory [7]. All procedures and operations in the field trial were conducted according to the code of Good Agricultural Practices that was issued by the Ministry of Agriculture of China and without the involvement of any endangered wildlife species or protected areas of land.

### 2.3 Exposure experiment

*X. laevis*, as an amphibian model organism [31], was selected for further exposure experiments. *X. laevis* tadpoles at stage 51 were selected for the TR exposure experiments at the real environmental levels (1 and 10 μg L^−1^) and ten-fold higher level (100 μg L^−1^) for 21 days, following the Nieuwkoop and Faber system [32]. Different concentrations of TR solution were prepared using DMSO. Both the control and exposure groups received identical concentration of DMSO (0.001%, v/v). Throughout the experiment, the tadpoles were maintained in 30 L glass tanks under stable conditions, with a 22 ± 1 °C temperature and a light–night cycle (12 h light, 12 h dark). The pH and conductivity of the exposure solution were maintained between 7.0 and 8.0 and 315–330 μS cm^−1^, respectively. In each glass tank, the dissolved oxygen concentration was maintained at or above 4 mg L^−1^, and the ammonia concentration was less than 0.2 mg L^−1^. The exposure media (20 L) was prepared using dechlorinated carbon-filtered water, and 50% of the tank volume was replaced with fresh exposure media daily to maintain TR concentrations. There were three replicates in each group, with 18 tadpoles per tank.

### 2.4 Phenotype measurements

During the exposure experiment, phenotype measurements, wet weight, total length, body length, and hindlimb length were recorded after exposure for seven days and the end of the exposure experiment (day 21), respectively. After the exposure experiment, the tadpoles were anesthetized with 175 mg L^−1^ MS-222 and rapidly frozen in liquid nitrogen, then stored at −80 °C for the subsequent experiment. The care and maintenance of the tadpoles adhered to the established guidelines following the Amphibian Metamorphosis Assay [33].

### 2.5 Locomotion assays

Locomotor activity was assayed with a Video-Track system (ViewPoint Life Science, Montreal, France) according to previously established methods [34,35]. The tadpoles were selected randomly and transferred into individual transparent culture tanks (150 × 150 mm) to measure the locomotor activity. After a 2-min acclimation period, the data of mobile frequency, mobile distance, and total movement tracking were collected per treatment for 10 min at ten frames per second, after which further data analysis was performed. Additionally, the locomotor behavior of tadpoles in the 0 μg L^−1^ group was chosen as a control.

### 2.6 Total community DNA extraction

The gut of tadpoles (three tissues per replicate) was performed to extract total community DNA with a FastDNA SPIN Kit for Soil (MP Biomedicals, USA) according to the manufacturer's instructions. The purity and concentration of DNA were measured by a NanoDrop ND-100 spectrophotometer (ALLSHENG, Hangzhou, China), and the quality was assessed by agarose gel electrophoresis. Total community DNA extraction was stored at −20 °C for follow-up processing.

### 2.7 Amplicon libraries preparation and sequencing

The V3–V4 region of the 16S rRNA gene fragments was amplified via polymerase chain reaction (PCR) following the Earth Microbiome guidelines [36]. PCR amplification of 16S rRNA gene fragments for microbiome studies was performed using the primers listed in Table S1. After quality control and adaptor ligation, equimolar DNA concentrations of each barcoded amplicon sample were subjected to next-generation sequencing on the Illumina HiSeq-PE250 platform.

### 2.8 Microbiome analyses

The acquired data were processed using QIIME2 v.2020.11.0 [37]. Cutadapt was employed to assess the quality and demultiplex the quality of paired-end reads. The microbiome of the gut of the control and treatment tadpoles was subjected to detailed profiling, with six replicates and 85,074–92,170 reads per sample group. After filtering and splicing all the samples, the clean tags were clustered, and chimeras were further removed for clustering into the Operational Taxonomic Unit (OTU) by UPARSE [38]. The representative sequences of the OTUs were selected and compared with the database to obtain species annotation information. Sequence counts ranged from 67,776 to 76,601 reads per sample, and we obtained a total of 328 bacterial OTUs at 97% identity after sequence processing and filtering the clean tags.

### 2.9 Isolation of intestinal bacteria

The gut of the tadpoles were ground into powder with liquid nitrogen, and sterilized ultrapure water was subsequently added. The sterilized ultrapure water was purified using a Milli-Q Water Purification System (Merck Millipore, Germany). After the mixture was settled, 1 mL of the supernatant was diluted to five concentration gradients. Subsequently, a fastidious anaerobe agar (FAA, Shanghai Rui Chu Biological Technology Co., Ltd., Shanghai, China) medium was then spread with 100 μL of the supernatant. The FAA medium was incubated in an AnaeroPack (Mitsubishi Gas Chemical America, New York, USA) containing 5% CO_2_ AnaeroPack·CO_2_ (Mitsubishi Gas Chemical America, New York, USA). Suitable culture conditions and media were selected to screen for specific bacteria based on microbiome analysis. According to the results of the morphological analysis, visible bacterial colonies were screened and streaked onto FAA media to isolate single colonies.

### 2.10 Identification of intestinal bacteria

Genomic DNA was extracted with a Takara MiniBEST Bacterial Genomic DNA Extraction Kit Ver.3.0 (TaKaRa Biotechnology, Japan) according to the manufacturer's instructions. The purity and concentration of the DNA were measured using a NanoDrop ND-100 spectrophotometer. The quality and the integrity of the DNA were checked by gel electrophoresis. For bacterial identification, we performed PCR amplification of 16S rRNA gene fragments using universal primers (Table S1). Subsequently, we conducted multiple alignments of the data and phylogenetic analysis of the sequences using MEGAX. A phylogenetic tree was constructed using the neighbor-joining method. Meanwhile, the alignment of the 16S RNA gene sequences of the isolated strains and the V3–V4 sequences of the OTU2 (*Fusobacterium*) and OTU12 (*Cetobacterium*) were consistent by Clustal X and GeneDoc software.

### 2.11 RNA-seq and transcriptome analysis

As directed by the manufacturers, the total RNA of tadpoles was extracted using a standard TRIzol reagent (Invitrogen, America). Oligh (dT) beads are used to remove rRNA and enhance mRNA. The enriched mRNA was fragmented into short fragments (200–300 bp). The first-strand of cDNA was reverse transcribed with random hexamers, and the second-strand cDNA was synthesized. Subsequently, the cDNA libraries were prepared from the total RNA of tadpoles and further sequenced on an Illumina novaseq 6000 platform. The raw data were cut for adapter sequence, and low-quality reads were filtered out to generate clean data using FASTP (https://github.com/OpenGene/fastp). With HISAT2 (http://ccb.jhu.edu/software/hisat2/index.shtml), the quality-filtered reads were mapped to the reference assembly accession (GCF_001663975.1), with a total of 34,034,694–38,678,970 mapped reads per sample. Fragments per kilobase per million fragments (FPKM) values were added, a pseudocount of 1, and the results were log_2_ transformed to calculate the gene expression. The log_2_ fold change was used to calculate the differences in fold changes between the control and treatment groups by the DESeq 1.32.0 package. Differentially expressed genes (DEGs) were identified based on the criteria of |log_2_ fold change| > 1 and false discovery rate (FDR) < 0.05. For further analysis, the DEGs were subjected to the enrichment analysis of KEGG pathways and network analysis visualization using Cytoscape (3.8.0).

### 2.12 Real-time quantitative polymerase chain reaction (RT-qPCR) analyses

To extract RNA from the intestinal tissues of tadpoles, a homogenate of three tissues per replicate was prepared on ice with TRIzol reagent. The extracted of RNA was subjected to reverse transcription using Primer-Script RT reagent kit (TaKaRa Biotechnology, Japan). Subsequently, RT-qPCR was conducted on the ABI 7500 real-time PCR system (Bioer Technology, Shanghai, China) following the manufacturer's instructions. The specific primers for RT-qPCR were designed using Primer Premier 5.0 software and the National Center for Biotechnology Information (NCBI) online tool, as shown in Table S1. To ensure that the input RNA in each sample was the same for RT-qPCR, the 18S ribosomal RNA (18S rRNA) gene was selected as intestinal reference genes. The 2^−△△Ct^ method was chosen to calculate the relative gene expression.

### 2.13 Quantification PCR of the microbial taxa

To quantify the relative abundance of *Fusobacterium* sp. and *Cetobacterium* sp. in the gut of tadpoles, FastDNA SPIN Kit for Soil (MP Biomedicals, USA) was used to extract total community DNA and specific primers (Table S1) was used to quantify the amplification of the 16S rRNA gene by qPCR. The qPCR was performed using ChamQ Universal SYBR qPCR Master Mix (Vazyme, China).

### 2.14 Colonization experiments of bacteria in the gut of tadpoles

*X. laevis* tadpoles at stage 51 were selected for the colonization experiments. After acclimatization to the environment, the tadpoles were randomly divided into three groups, each equally divided into three replicates with 18 tadpoles per tank. The diet preparation was performed as described previously with appropriate modifications [39]. The experimental groups were administered *Fusobaterium* and *Cetobacterium* feeds, respectively. Only sterilized feeds were fed to the control group. Throughout the experiment, the exposure media (20 L) was prepared using sterilized dechlorinated carbon-filtered water, and 50% of the tank volume was replaced with fresh exposure media daily. Sterilized water was detected that there were no bacteria in it.

### 2.15 Statistical analyses

The data were analyzed via One-way Analysis of Variance (ANOVA) and student's *t*-test analysis (*p* < 0.05) to evaluate statistical significance. Version 26.0 of the Statistical Package for the Social Sciences (SPSS) was used to evaluate and calculate the significance of the differences between the control and treatment groups. Cytoscape (3.8.0) was used for network visualization. Alpha diversity and principal coordinate analysis (PCoA) analyses were performed on Tutools plantform (https://www.cloudtutu.com).

### 2.16 Data availability

All the raw sequence data have been deposited in the Sequence Read Archive of NCBI. The gut microbiome data of tadpoles was deposited under the accession PRJNA910976. Transcriptome datasets were deposited under accession PRJNA910979. All other raw data for all figures and tables are available from the corresponding authors upon reasonable request.

## 3 Results

### 3.1 Impaired development of the host upon exposure to the biotoxin

To investigate the effect of TR on frog development, we measured a series of fundamental development indexes, including the total length, hindlimb length, body length, and wet weight of tadpoles exposed to TR at the real environmental levels (1 and 10 μg L^−1^) and at a ten-fold higher level (100 μg L^−1^). After seven days of TR exposure, there was a downward trend (the *p* values for total length, body length, hindlimb length, and weight were 0.334, 0.777, 0.051, and 0.284, respectively) in the 1 μg L^−1^ group compared with those in the control group (in the absence of TR) (Fig. S4). After further continuous observation until the end of the exposure experiment, compared with the control group, we observed statistical differences in the development indexes between the control and the TR-exposed groups at both 1 and 10 μg L^−1^ TR. In the control group, the total length of tadpoles measured 7.10 ± 0.33 cm, while it dose-dependently decreased to 6.09 ± 0.50 and 5.50 ± 0.79 cm under 1 and 10 μg L^−1^ of TR, respectively (Fig. 1b). Interestingly, an increase in total length was observed in the tadpoles upon exposure to 100 μg L^−1^ TR in comparison with those upon exposure to 1 or 10 μg L^−1^ TR, but the difference was not statistically significant compared with that in the control group (Fig. 1b).

TR exposure induced a similar pattern in the total length, hindlimb length, and wet weight of the tadpoles (Fig. 1b–d). Furthermore, the body length of the 1 and 10 μg L^−1^ groups was reduced compared with the control group, while the body length of the 100 μg L^−1^ group was not significantly different from the control group (Fig. 1e). Compared with that in the control group, there was a developmental delay observed in the treatment groups (Table S2). These results indicated that exposure to TR at the environmental levels could imparied the development of tadpoles.

### 3.2 Host locomotor activity increases upon exposure to TR

In the TR exposure experiment (equivalent to the environmental exposure level), the behavior of the tadpoles changed as well, and their swimming speed showed a visually increasing trend in contrast to the control group. Because locomotor activity is also a sensitive indicator of developmental toxicity, we conducted a precise behavioral assay. As a result, we found that exposure to 1, 10, and 100 μg L^−1^ TR significantly increased the average swimming speed of the tadpoles. Specifically, the swimming speed of the tadpoles exposed to 1, 10, and 100 μg L^−1^ TR significantly increased by 49.96%, 84.83%, and 57.58%, respectively (Fig. 1f).

Moreover, compared with the control group, tadpoles exposed to 1, 10, and 100 μg L^−1^ concentrations of TR showed 3.11-, 4.71-, and 3.74-fold increases in average activity, respectively (Fig. 1g). The locomotor activity of the tadpoles increased for the entire tracking duration of 10 min (Fig. 1h) or every 10 s (Fig. S5). Interestingly, TR at 1–100 μg L^−1^ did not exert a dose-dependent impact on either speed or activity (Fig. 1f and g), and the maximum impact of TR was observed at the level equivalent to the environmental exposure level (Fig. 1f and g). These results indicate that the behavior of tadpoles exposed to TR at the environmental concentration levels was drastically altered, characterized by increased locomotor activity.

### 3.3 Host developmental disorders are associated with a shift in the gut symbiome to the pathobiome

**Fig. 2:**
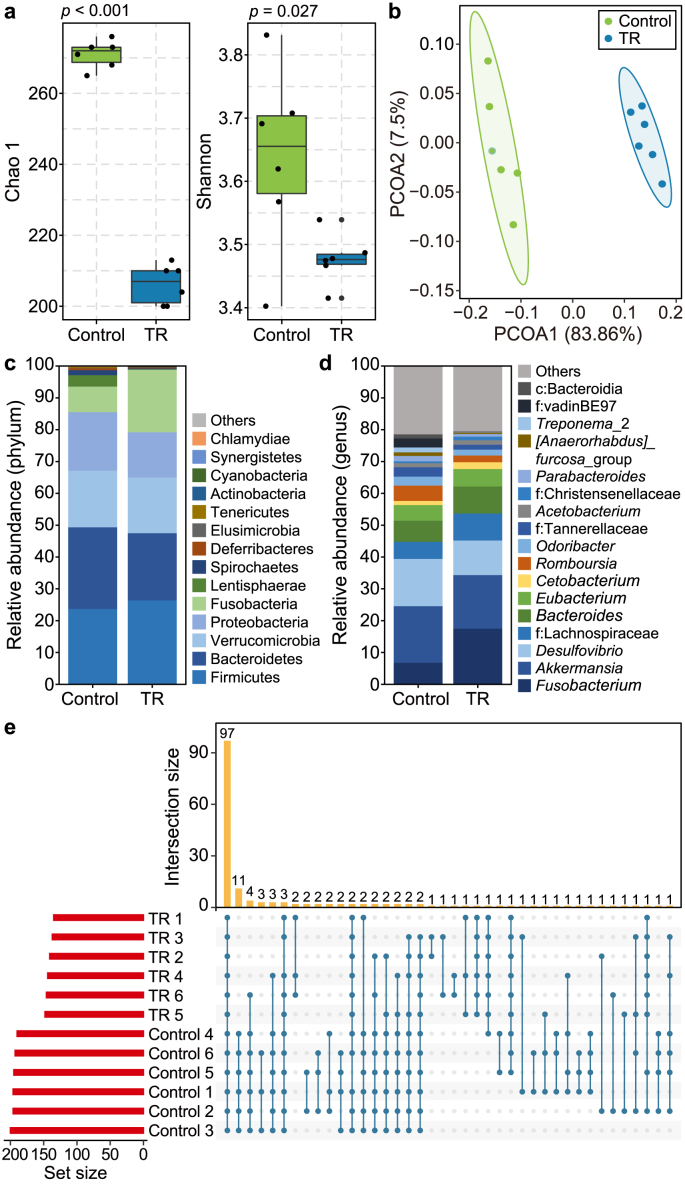
**Characterization of the gut microbial community in the biotoxin-exposed tadpoles. a–b,** Analysis of the gut microbial community structure based on alpha (**a**) and beta (**b**) diversities. Significant differences were observed between the control (TR at 0 μg L^−1^) and TR-exposed groups (10 μg L^−1^). *n* = 6 replicates. **c**–**d,** Comparison of the gut microbial community composition between the control (TR at 0 μg L^−1^) and TR-exposed group (10 μg L^−1^) at the phylum (**c**) and genus (**d**) levels. *n* = 6 replicates. c and f indicate unassigned taxa at the taxonomic levels of class and family, respectively. **e,** UpSet plot of the gut microbial community in the TR-exposed (10 μg L^−1^) tadpoles at the genus level. The length of the red bars (bottom left) indicates the total size sets of the genera. The blue symbols, connected with blue lines, represent the intersections between the sets and the number. The yellow columns indicate the frequency of these intersections.

To investigate whether the gut microbiome changed in the development-impaired hosts, we further analyzed the community structure and composition of the gut microbiome. Analysis of alpha diversity, including the Chao 1 richness index and Shannon index, showed that the abundance and diversity of the gut microbiome in tadpoles exposed to TR (10 μg L^−1^) were significantly lower than those in the control group (Fig. 2a). Using beta diversity-based PCoA, we observed a significant difference in community composition between the control and exposure groups (Fig. 2b, Dataset 2). In addition, through a rank-abundance curve, it was found that the control group had higher species abundance and more uniform species composition than the TR-exposed groups (Fig. S6a). Remarkably, the exposure group had a higher proportion of dominant bacteria, and the diversity of species was lower than that of the control group (Fig. S6b).

We further analyzed the composition of the microbial community in the control and exposure groups. At the phylum level, the gut microbiome of tadpoles in the control and exposure groups was dominated by Bacteroidetes (mean, 25.70% and 21.05% in the control and exposure groups, respectively) and Firmicutes (mean, 23.72% and 26.49% in the control and exposure groups, respectively) (Fig. 2c, Fig. S7a and b, Table S3). Consequently, the further detailed resolution analysis revealed a distinct gut microbial composition in the exposure group (Fig. S8a and b, Fig. S9, Dataset 3), with a marked change at the genus level (Fig. 2d and e). This indicates that exposure to TR results in a shift from a healthy intestinal microbiome to a pathobiome in tadpoles.

**Fig. 3:**
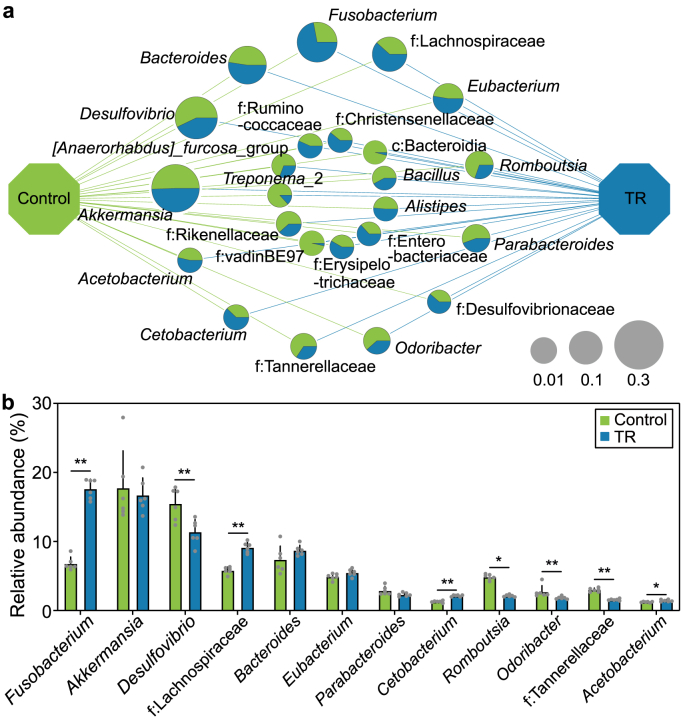
**Identification of core taxa of the gut pathobiome in biotoxin-exposed tadpoles. a**, Co-occurrence network of the microbial community of the gut pathobiome in the tadpoles upon exposure to 10 μg L^−1^ TR. Nodes indicate the top 20 most abundant taxa in each treatment, with node size indicating relative abundance. The pie graphs at nodes show the proportion of each treatment, and labels demonstrate taxa. c, o, and f indicate unassigned taxa at the taxonomic levels of class, order, and family, respectively. **b,** Analysis of the relative abundance of the core taxa of the gut pathobiome in the tadpoles. Student's *t*-test (two-tailed). ∗*p* < 0.05, ∗∗*p* < 0.01. The values are presented as the means ± s.d. (shown as error bars, *n* = 6).

To infer the potential core taxa of the pathobiome, a co-occurrence network of microbial communities in the tadpoles’ gut was visualized (Fig. 3a). We defined the taxa with relative abundances higher than 0.01 as the core taxa of the gut microbiome. The network linked 12 core taxa, including *Fusobacterium*, *Cetobacterium*, *Parabacteroides*, *Eubacterium*, *Bacteroides*, *Desulfovibrio*, *Akkermansia*, *Odoribacter*, *Romboutsia*, *Acetobacterium*, Lachnospiraceae, and Tannerellaceae (Fig. 3a), among which *Acetobacterium, Cetobacterium*, *Fusobacterium*, and Lachnospiraceae were inferred as the core taxa of the pathobiome due to their significant enrichment in the TR-exposed group (Fig. 3b–Table S4.

### 3.4 Core pathobiome taxa mediate the TR-induced developmental disorders in the host

**Fig. 4:**
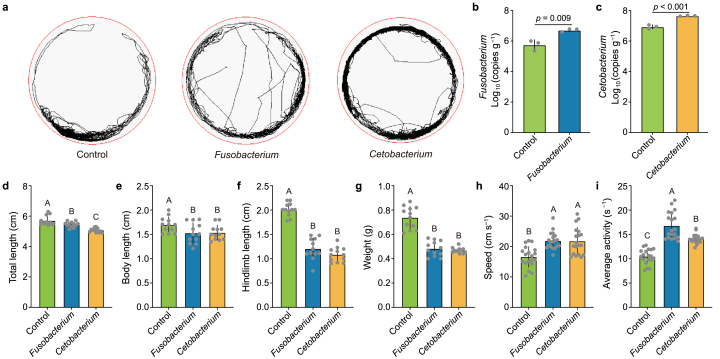
**Core gut pathobiome taxa responsible for the altered development and locomotor activity of the biotoxin-exposed tadpoles. a**, Whole tracking of tadpoles recorded for 10 min is shown for the control group and those transplanted with *Fusobacterium* or *Cetobacterium* groups. **b**–**c,** Quantitative analysis of the colonization of *Fusobacterium* (**b**) and *Cetobacterium* (**c**) in the gut of the tadpoles after microbial transplantation. *n* = 3 replicates. **d–g,** Effects of the transplantation of *Fusobacterium* and *Cetobacterium* on the total length (**d**), body length (**e**), hindlimb length (**f**), and weight (**g**) of the tadpoles. *n* = 12 replicates. **h**–**i,** Effects of the transplantation of *Fusobacterium* and *Cetobacterium* on the swimming speed (**h**) and average activity (**i**) of the tadpoles. *n* = 19 for each. Different letters with error bars indicate a significant difference based on ANOVA with LSD test (*p* < 0.05).

To untangle the causality between the TR-induced developmental impairment and increased locomotor activities in tadpoles and the shift of the symbiome toward a pathobiome, we conducted a preliminary comparison involving the tadpoles fed isolated bacteria in the absence of TR. Using culture-dependent isolation, we obtained 154 bacterial isolates from the gut of developmentally disordered tadpoles. Interestingly, only 16 bacterial isolates had effects similar to those observed under TR-induced excitability (Fig. 4a, Fig. S10). Phylogenetic analysis based on the 16S rRNA sequence showed high homology with *Fusobacterium* sp. (ten isolates) and *Cetobacterium* sp. (six isolates) (Figs. S11 and 12), respectively. Furthermore, the 16S rRNA gene sequences of these isolates were consistent with the core pathobiome taxa identified in the microbiome profiling (Figs. S13 and 14, Dataset 4).

To clarify whether TR directly affects the tadpoles or indirectly affects them through the gut pathobiome, the tadpoles were fed with the inferred core taxa in the gut pathobiome. After quantification polymerase chain reaction (qPCR) confirmed the successful colonization of *Fusobacterium* and *Cetobacterium* in the intestinal tissue (Fig. 4b and c). Further precise quantitative analysis revealed that the total length of the tadpoles fed *Fusobacterium* and *Cetobacterium* were 5.50 ± 0.18 and 5.08 ± 0.14 cm, respectively, which were lower than that of in contrast with the control group (5.71 ± 0.31 cm) (Fig. 4d). Similarly, there was a marked decrease in the body length, hindlimb length, and wet weight of the tadpoles in the treatment groups (Fig. 4e–g). In addition, we found that the average swimming speed and average activity of tadpoles increased in the groups fed *Fusobacterium* and *Cetobacterium*. Specifically, the average speed of the tadpoles increased obviously by 31.78% and 31.09%, respectively (Fig. 4h), and excitability was significantly induced (Fig. 4i). These results together indicate that the developmental disorders induced by TR were mediated by the core pathobiome taxa inhabiting the tadpoles’ gut.

### 3.5 The gut pathobiome core taxa-reprogrammed transcriptome underlies host developmental and behavioral disorders

**Fig. 5:**
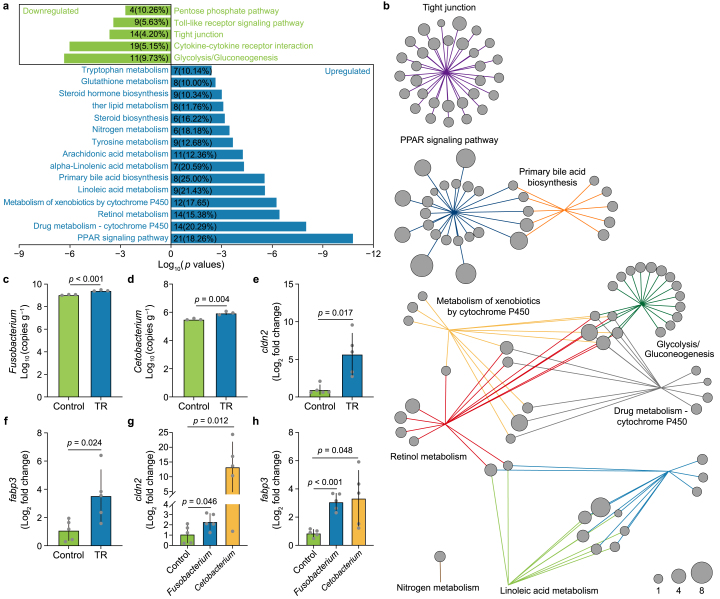
**Transcriptome reprogramming in the gut pathobiome-harboring tadpoles. a**, KEGG pathways with enrichment of genes whose expression was significantly upregulated and downregulated genes in the TR-treated group (10 μg L^−1^), respectively. The number on the *x*-axis represents the *p* values of the enrichment of the KEGG pathway by log_10_-transformed. The quantity of DEGs in each enrichment pathway is exhibited by the numbers in the plot, and the proportion of all genes is also indicated in the pathway (in parentheses). **b,** Network analysis of the DEGs associated with the top ten KEGG enrichment pathways. The circle size denotes the relative fold change (log_2_-transformed) of gene expression. **c**–**d,** The colonization of *Fusobacterium* (**c**) and *Cetobacterium* (**d**) in the gut of the tadpoles upon exposure to 10 μg L^−1^ TR. *n* = 3 for replicates. **e**–**f,** The expressions of gene *cldn2* (**e**) and *fabp3* (**f**) in the TR-exposed tadpoles (10 μg L^−1^). *n* = 5 for replicates. **g**–**h,** The gene *cldn2* (**g**) and *fabp3* (**h**) expressions in the tadpoles fed with *Fusobacterium* and *Cetobacterium*, respectively. *n* = 5 for replicates. The values are presented as the means ± s.d. Student's *t*-test (two-tailed), *p* values were shown on the top of the paired columns.

We further explored the global gene expression in the development-disordered tadpoles by transcriptomic analyses. Each pair of groups under the same treatment had an *r*-value of 1.00 in the heatmap with correlation coefficient analysis (Fig. S15a). The data repeatability for subsequent analysis was indicated by an *r* value of 0.9923 for log_2_-transformed normalized gene expression between the control and treatment groups by a linear regression analysis (Fig. S15b). Each sample's gene body coverage profile demonstrated no bias in the control or treatment groups (Fig. S16a-f). The principal component analysis (PCA) of the RNA sequencing revealed that the TR-exposed samples significantly differed from those not exposed to TR (Fig. S17a). The UpSet plot and volcano plot illustrated the significant differences in gene expression between the control and treatment groups (Fig. S17b and c). Furthermore, compared with the control group, there were alterations in the expression of 1403 genes in the TR treatment group based on RNAseq analysis; among them, 961 genes were significantly upregulated (log_2_-transformed fold change >1, *p* < 0.05) (Datasets 5), while 442 genes were significantly downregulated (log_2_-transformed fold change < −1, *p* < 0.05) (Dataset 6). We used all the DEGs in the top 10 KEGG pathways to generate a column clustering heatmap, showing obvious gene expression differences between the control and treatment groups (Fig. S18, Dataset 7). The up- and down-regulated genes were further subjected to KEGG pathway enrichment analysis. We found 75 pathways enriched by significantly downregulated genes, five of which were significantly enriched (Fig. 5a, Dataset 8). In contrast, 115 pathways were enriched by upregulated genes, among which 15 pathways were significantly enriched (Fig. 5a, Dataset 9). A scatter plot of the enriched KEGG pathways showed 16 significantly enriched pathways among all DEGs (Fig. S19, Dataset 10).

The DEGs enriched in the top ten KEGG pathways were subjected to network analysis (Fig. 5b, Dataset 11). The major clusters were the peroxisome proliferator-activated receptor (PPAR) signaling pathway and tight junction (TJs), while the remaining clusters were related to metabolism. The high induction of a set of PPAR and TJ genes was associated with a dramatic increase in the population density of *Fusobacterium* and *Cetobacterium* (Fig. 5c and d). These genes, included claudin 2 L homeolog (*cldn2*) and fatty acid binding protein 3 L homeolog (*fabp3*), were induced which are associated with the tight junction pathway and PPAR signaling pathway, respectively (Fig. 5e and f, Dataset 12). Through RT-qPCR, we further found that PPAR (*fabp3*) and TJ (*cldn2*) gene expressions were both induced by the transplantation of either *Fusobacterium* or *Cetobacterium*, verifying the causative impact of *Fusobacterium* and *Cetobacterium* on the induction of PPAR (*fabp3*) and TJ (*cldn2*) gene expression (Fig. 5g and h). These results together profiled the gut pathobiome-triggered transcriptome reprogramming that underlies developmental and behavioral disorders in the host.

## 4 Discussion and conclusion

As climate change continues, there is a growing concern that the prevalence of phytopathogens and the associated biotoxin pollution in aquatic environments may persist or worsen [2,5]. For a long time, research on biotoxin pollution and other environmental pollutants (intentionally or unintentionally introduced into the environment) has focused on the direct harm to exposed plant and animal communities. Recently, a pioneering study shed light on the disruption of the normal metabolic rhythm of the gut microbiome in host mammals with a high-fat diet, indicating a potential link between the gut pathobiome and diseases caused by extrinsic factors [40]. A study suggested that the gut pathobiome could exacerbate systemic inflammation and behavioral disorders in the neurological disease CADASIL [41]. However, the causality between the gut pathobiome and host diseases, especially upon exposure to emerging biotoxin pollution, remained largely unexplored and elusive. Based on the model amphibian organism *X. laevis*, we uncovered a previously unknown causal role of the gut microbiome in frog population decline attributed to biotoxin pollution. In this study, the biotoxin TR was found to drive the assembly of the gut pathobiome, which further led to locomotor excitability and developmental disorders in the tadpoles.

In animals, locomotor activity is critical in growth and development, which was previously reported to be modulated by the gut microbiome [42,43]. Our current study found that increased locomotor activity (Fig. 1f and g) and induced developmental disorders (Fig. 1b–e) were present in the tadpoles upon exposure to the biotoxin TR. Furthermore, it was highlighted that the gut microbiome was markedly altered in development-disordered tadpoles upon TR exposure, in which *Acetobacterium, Cetobacterium, Fusobacterium*, and Lachnospiraceae were identified as significantly enriched taxa in the pathobiome (Fig. 2, Fig. 3). To investigate whether TR affects tadpoles directly or indirectly through the gut pathobiome, we performed a colonization experiment by feeding tadpoles bacterial isolates corresponding to the inferred gut pathobiome taxa. In the absence of TR, *Fusobacterium* and *Cetobacterium* similarly caused developmental disorders and increased locomotor activity (Fig. 4), suggesting that they are the causal core taxa of the gut pathobiome. A variety of clinical presentations identified the genus *Fusobacterium* as a causative agent in serious diseases, such as colitis and periodontal disease [[44], [45], [46]]; while the genus *Cetobacterium* was also reported as a possible pathogen in fish species, such as Nile tilapia, zebrafish [47], and colorectal cancer in humans [48]. Our study has shed light on the causality of these core taxa in the gut pathobiome, which triggered the excitability of locomotor activity and developmental disorders.

**Fig. 6:**
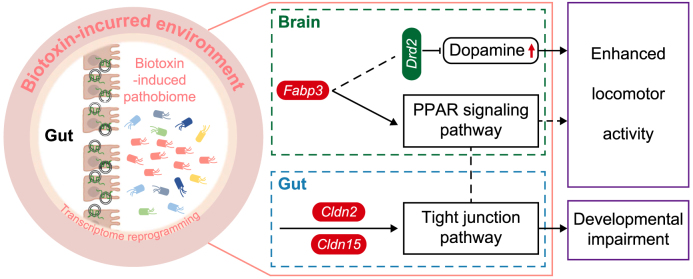
**Proposed schematic illustration of developmental and behavioral disorders mediated by biotoxin exposure-driven assembly of the gut pathobiome**. The left panel of the figure shows that the biotoxin TR exposure drives the shift of the normal gut microbiome to the pathobiome, which causes developmental and behavioral disorders in the tadpoles. The detailed mechanism underlying the gut pathobiome-mediated disorder is shown in the right panel of the figure. In the reprogrammed transcriptome, a series of genes in the gut–brain axis involving *fabp3*, *cldn2*, *cldn15*, and *drd2*, differentially express and trigger the excitability of locomotor activity and morphogenesis impairment in tadpoles. The upregulated genes are indicated with red ellipses, and the downregulated genes are denoted with green. The red arrow demonstrates an increased level of dopamine.

By transcriptome profiling-guided analysis, we explored the mechanism underlying the disordered activities and development triggered by the pathobiome, and observed a significant enrichment of the PPAR signaling pathway along with the TR-induced pathobiome (Fig. 5a,b, Fig. S19). PPARs can influence the gut mucosal homeostasis and are pivotal in modulating host pathogenesis by influencing the gut and whole-body immune response [49]. Interestingly, PPARs also engage in binary crosstalk with the gut microbiome [50]. For instance, Firmicutes and Fusobacteria were potent activators of PPARs in the gut [50], while the dysregulation of PPARs was involved in the dysbiosis of the gut microbiome in *Trichinella spiralis*-infected mice [51]. Further analysis revealed *fabp3*, a representative PPAR gene expressed in the brain, was markedly enriched in both the TR- and pathobiome-exposed groups (Fig. 5f–h), along with the accumulation of dopamine (DA) (Fig. S20) and the downregulation of D2 dopamine receptor (*drd2*) B isoform X3 (Dataset 13). Activation of *drd2* generally decreases dopamine release and further reduces locomotor activity, and dopamine-related locomotor activity is controlled by the binding of *fabp3* to *drd2* in the brain [52,53]. Hence, these results indicated that the core taxa of the gut pathobiome induced the promotion of locomotor activity, possibly through the tuning of the *fabp3*-associated dopamine and PPAR signaling pathways through the gut–brain axis (Fig. 6).

Moreover, along with the TR-induced pathobiome, we also noticed that the TJ pathway was enriched in the transcriptome network (Fig. 5b). In fact, some TJ protein-coding genes, such as *claudin 3,* are reported to be transcriptionally regulated by PPARs [54]. Tight junctions are crucial in maintaining epithelial barrier function [55] and are composed of cytoplasmic plaque proteins and integral transmembrane proteins. These proteins include zonular occludens (ZOs), which link the TJs to the cytoskeleton and occludin, junctional adhesion molecules (JAMs), and claudins, all of which define the properties of the paracellular pore [56]. Some claudins, such as claudin-2, are known to form pores that allow specific ions to pass preferentially, while others, such as claudin-1, -3, -4, -5, and -7, prevent the formation of specific ions and enhance the intestinal barrier [[57], [58], [59]]. An increase in paracellular permeability is a causal factor of chronic diseases, such as inflammatory bowel disease (IBD) [60]. A previous study suggested that claudin-2 expression was elevated in IBD and IBD-associated dysplasia [61]. Extracellular vesicle-derived claudin-2 serves as a biomarker to predict colorectal cancer liver metastases in humans [62]. The gut microbiome can upregulate the expression of *cldn2* (claudin-2) in the gut of mice by secreting Staphylococcal enterotoxin B (SEB) and cholera toxin (CT) [63]. Interestingly, we observed that *cldn2* (claudin 2 L homeolog) was upregulated in the TR- and pathobiome-exposed groups (Fig. 5e–g, Dataset 13). This is the possible reason that the genera *Fusobacterium* and *Cetobacterium* induce inflammation in the gut by regulating *cldn2*.

In addition, claudin-15 (*cldn15*) is an essential regulator of paracellular Na^+^ flux and transcellular nutrient absorption [64,65], and is upregulated in celiac disease (CD) [64]. Previous studies have demonstrated that the dysfunction of tight junctions (TJs) caused by disordered expression of *cldn2* and *cldn15* is involved in the pathogenesis of diarrheal diseases [58,66,67]. Diarrheal diseases are characterized by increased gut permeability, regulated by TJ proteins [68]. Diarrheal diseases or malabsorptive diseases are leading causes of malnutrition and can even lead to death in severe cases. Our study found that the expressions of *cldn2* and *cldn15* were significantly upregulated in the TR-exposed tadpoles. These results indicate that dysfunction of the TJ pathway, induced by the core taxa of the gut pathobiome in tadpoles, can result in malnutrition and further developmental impairment (Fig. 6).

In the past decade, biotoxin pollution has emerged as a growing global concern, impacting public health and potentially affecting frog populations. In our current study, we have discovered the role of the gut pathobiome in mediating locomotor activity and developmental impariments in frogs exposed to environmental biotoxin pollution. Additionally, our research has revealed that two core pathobiome taxa, *Fusobacterium* and *Cetobacterium*, can directly disrupt frog health even without biotoxin exposure, potentially by interfering with critical pathways in the gut–brain axis. These present findings not only emphasize the gut pathobiome as the endogenous trigger for the toxicity of exogenous pollutants, but also provide a fresh perspective on the ongoing decline of frog populations in the context of global change.

## Ethics approval and consent to participate

All animal procedures were approved by the Experimental Animal Ethics Committee of Zhejiang University.

## CRediT authorship contribution statement

**Qianqian Pan:** Conceptualization, Data Curation, Formal Analysis, Methodology, Software, Visualization, Writing - Original Draft. **Tianxing Lv:** Data Curation, Investigation. **Haorong Xu:** Data Curation, Investigation. **Hongda Fang:** Data Curation. **Meng Li:** Methodology. **Jiaping Zhu:** Investigation, Methodology. **Yue Wang:** Investigation. **Xiaoyan Fan:** Methodology. **Ping Xu:** Investigation. **Xiuguo Wang:** Investigation. **Qiangwei Wang:** Conceptualization, Methodology. **Haruna Matsumoto:** Conceptualization, Software, Supervision, Writing - Review & Editing. **Mengcen Wang:** Conceptualization, Formal Analysis, Funding Acquisition, Project Administration, Writing - Review & Editing.

## Declaration of competing interest

The authors declare that they have no known competing financial interests or personal relationships that could have appeared to influence the work reported in this paper.
