# Supplementary material for: Gut pathobiome mediates behavioral and developmental disorders in biotoxin-exposed amphibians
Source: Environ Sci Ecotechnol. 2024 Mar 21;21:100415. doi: 10.1016/j.ese.2024.100415 (PMC10992726; doi:10.1016/j.ese.2024.100415)
Supplement: Multimedia component 1 [file mmc1.docx]

**Supporting Information**

**Gut Pathobiome Mediates Behavioral and Developmental Disorders in Biotoxin-exposed Amphibians**

Qianqian Pan^a,b^, Tianxing Lv^a,b^, Haorong Xu^a,b^, Hongda Fang^a,b^, Meng Li^b^, Jiaping Zhu^b^, Yue Wang^a,b^, Xiaoyan Fan^a,b^, Ping Xu^c^, Xiuguo Wang^d^, Qiangwei Wang^a,b^, Haruna Matsumoto^a,b^*, Mengcen Wang^a,b,e^*

^a^ Ministry of Agricultural and Rural Affairs Laboratory of Molecular Biology of Crop Pathogens and Insects, Zhejiang University, Hangzhou 310058, China.

^b^ Institute of Pesticide and Environmental Toxicology, College of Agriculture and Biotechnology, Zhejiang University, Hangzhou 310058, China.

^c^ Institution of Tea Science, Zhejiang University, Hangzhou 310058, China.

^d^ The Tobacco Research Institute, Chinese Academy of Agricultural Sciences, Qingdao 266101, China.

^e^ Global Education Program for AgriScience Frontiers, Graduate School of Agriculture, Hokkaido University, Sapporo, Japan.

Corresponding authors’ email addresses: [mharuna@zju.edu.cn](mailto:mharuna@zju.edu.cn); [wmctz@zju.edu.cn](mailto:wmctz@zju.edu.cn).

* To whom correspondence should be addressed.

**Supporting Information**

**Number of pages: 30**

**Number of tables: 4**

**Number of figures: 20**

**Number of datasets: 12**

**Table list**

**Table S1. Oligonucleotide primers for bacterial isolation and identification.**

**Table S2. The developmental stages of TR in tadpoles upon exposure to TR for 21 days.**

**Table S3. Dominant bacterial phyla of the gut microbiota in tadpoles at different groups.**

**Table S4. Percentage of the core taxa in the gut microbiota of tadpoles between the control and TR-treated groups (10 μg L^−1^).**

**Dataset list**

**Data 1. The relationship of the frogs population and tropolone levels in region-specific rice paddy.**

**Data 2. Beta diversity based on PCoA with Bray-Curtis dissimilarity matrix in the control group and the tropolone-exposed group.**

**Data 3. The shared OTUs in control and tropolone groups.**

**Data 4. The DNA sequence alignment of *Fusobacterium* and *Cetobacterium.***

**Data 5. List of significantly upregulated genes following tropolone treatment.**

**Data 6. List of significantly downregulated genes following tropolone treatment.**

**Data 7. The all differentially expressed genes of the top 10 KEGG pathways.**

**Data 8. Enriched KEGG pathways with significantly downregulated genes.**

**Data 9. Enriched KEGG pathways with significantly upregulated genes.**

**Data 10. Significantly enriched KEGG pathways with DEGs.**

**Data 11. All differentially expressed genes of the top 10 KEGG pathways.**

**Data 12. The associated genes of the molecular mechanism.**

**Figure list**

**Fig. S1. The chemical structure of biotoxin tropolone (TR).**

**Fig. S2. The density of frogs is negatively correlated with TR in paddy fields.** Heatmap of Pearson’s correlation coefficients. Asterisk (***) indicates a significant correlation at *p* < 0.001.

**Fig. S3. The flow chart of the field sampling and the simplified workflow of subsequent analysis. a–b,** Field sampling in paddy fields. The chessboard-type sampling was applied to calculate the population of frogs in 100 plants per paddy field. The five-point sampling method was implemented to collect and mix the paddy water. The samples were transported to the Laboratory. **c,** The subsequent experiment and analysis. The correlation analysis between frog populations and TR concentrations.

**Fig. S4. Alterations of development in the tadpoles upon exposure to TR at seven days. a–d**, Effects of TR exposure (0, 1, 10, 100 μg L^−1^) on the total length (a), body length (b), hindlimb length (c), and weight (d) of tadpoles. *n* = 6 for replicates. Above all have the different letters with error bars, which indicate a significant difference by one-way analysis of variance (ANOVA) with LSD test (*p* < 0.05).

**Fig. S5. The representative individual tracking of tadpoles in the control (TR at 0 μg L^−1^) and the TR-exposed groups (TR at 1, 10, 100 μg L^−1^).** The tracking of tadpoles was recorded every 10 seconds.

**Fig. S6. Overall structure of the gut microbiota of tadpoles upon exposure to 10 μg L^−1^ concentration of TR. a,** Rank-abundance curve of microbial species in each sample (*n* = 6). **b,** Rarefaction curves of 16S rRNA in samples of tadpoles in the control and TR-exposed groups (*n* = 6).

**Fig. S7. The boxplot of the dominated phyla of the tadpoles’ gut microbiome in the control (TR at 0 μg L^−1^) and the TR-exposed (10 μg L^−1^) groups. a–b,** The boxplots of phyla Firmicutes (**a**) and Bacteroidetes (**b**). *n* = 6 replicates.

**Fig. S8. Bacterial composition of the gut microbiome in tadpoles.** The stacked histograms illustrate the class-level (**a**) and family-level (**b**) bacterial composition of the gut microbiome of tadpoles in the control (TR at 0 μg L^−1^) and the TR-exposed (10 μg L^−1^) groups.

**Fig. S9. UpSet plot of the gut microbiome in the TR-exposed (10 μg L^−1^) tadpoles.** The length of the red bars (bottom left) indicates the total size sets of the OTUs. The blue symbols connected with blue lines represent the intersections between these sets and the number, while the yellow columns indicate the frequency of these intersections. *n* = 6 replicates.

**Fig. S10. The representation of individual tracking of tadpoles in the control groups and the transplantation of core taxa bacteria groups.** The tracking of tadpoles was recorded every ten seconds.

**Fig. S11. Phylogenetic analysis of the 16S rRNA gene sequence of *Fusobacterium* sp.** The phylogenetic tree was constructed by the Neighbor-joining method using MEGA, with the lengths of the horizontals proportional to the differences.

**Fig. S12. Phylogenetic analysis of the 16S rRNA gene sequence of *Cetobacterium* sp.** The phylogenetic tree was constructed by the Neighbor-joining method using MEGA, with the lengths of the horizontals proportional to the differences.

**Fig. S13. Sequence alignment of *Fusobacterium* strains.** Fuso indicates the 16S rRNA gene sequence of *Fusobacterium* sp., isolated from the gut of tadpoles. The sequence of OTU2 is obtained from the microbiome profiling.

**Fig. S14. Sequence alignment of *Cetobacterium* strains.** Ceto indicates the 16S rRNA gene sequence of *Cetobacterium* sp., isolated from the gut of tadpoles. The sequence of OTU12 is obtained from the microbiome profiling.

**Fig. S15. Reliability testing for the transcriptome analysis of TR-treated (10 μg L^−1^) tadpoles. a,** The colors of plots display the degree between the control and TR exposure groups, and the number of the plots indicates the correlation coefficient *r* values. **b,** The light blue line in the plot represents the simple linear regression analysis of the values between the log_2_-transformed normalized gene expressions of the control (control 1, control 2, and control 3) and the TR-exposed (TR 1, TR 2, and TR 3) samples. For each transcriptome pair, Pearson’s correlation coefficients (R^2^ = 0.9847) indicated the data repeatability (*p* < 0.001, Student’s *t*-test, two-tailed).

**Fig. S16. Gene body coverage profile for each sample. a–c,** Gene body coverage profile for control groups. **d–f**, Gene body coverage profile for TR-exposed groups.

**Fig. S17. Analysis of the differentially expressed genes in tadpoles upon exposure to 10 μg L^−1^ concentration of TR. a,** PCA analysis of RNAseq. *n* = 3 replicates. **b,** UpSet plot demonstrates the genes of tadpoles are significantly changed in tadpoles upon exposure to TR. The length of the red bars (bottom left) indicates the total size sets of the differentially expressed genes. The blue symbols connected with blue lines represent the intersections between the sets and the number, while the yellow columns indicate the frequency of these intersections. *n* = 3 replicates. **c,** Transcriptome pattern in tadpoles upon exposure to TR. The red dots represent the up-regulated genes, while down-regulated genes are shown in blue. The grey dots in the center are not significantly altered.

**Fig. S18. The cluster heatmap of the difference expression genes of the top 10 KEGG pathways in tadpoles upon exposure to TR (10 μg L^−1^).** Hierarchical clustering of 108 genes that are differentially expressed in the RNAseq. A fitted generalized linear model products heatmap that shows fold changes derived from log_2_-transformed fold changes in the treatment groups compared with the control groups.

**Fig. S19. The scatter plot of the top 16 significantly enriched KEGG pathways in tadpoles upon exposure to 10 μg L^−1^ concentration of TR.** The rich factor represents the ratio of the DEGs number to the total gene number in a certain pathway. The size and color of the dots indicate the number of DEGs mapped to the related pathways and the range of the *p* value of KEGG pathways enrichments by -log_10_-transformed, respectively.

**Fig. S20. Determination of dopamine in the tadpoles upon exposure to 10 μg L^−1^ concentration of TR.** The concentration of dopamine was detected by optical density at 450 nm.

**Tables**

**Supplementary Table S1. Oligonucleotide primers for bacterial isolation and identification.**

| Gene/Primer names | Forward primer (5’-…-3’) | Reverse primers (5’-…-3’) | Purpose/Function |
| --- | --- | --- | --- |
| 16S rRNA V3-V4 | ACTCCTACGGGAGGCAGCA | GGACTACHVGGGTWTCTAAT | Microbiome profiling of bacterial comm. |
| 16S rRNA | TTCCGGTTGATCCTGCCGGA | AAGGAGGTGWTCCARCC | Identification of bacteria |
| *18S* | AACGGCTACCACATCCAAGG | CACCAGACTTGCCCTCCAAT | Reference gene in tadpoles |
| *Cldn2.L* | CGGCACCATTCTCTGTACTTC | CGCTTGGAGGTCTCTGTTATG | Claudin 2 L homeolog |
| *Fabp3.L* | TGGAGAAATTTGCAGGAACC | ACTACGATTTTGTCCCCATCC | Fatty acid binding protein 3 L homeolog |
| Fuso | CCGTCACACCACGAGAGTT | TAGGACGACGCATACCCTC | Quantification of *Fusobacterium* |
| Ceto | GCTTGCCGGAACTTAGT | TCATCGCAGGCAGTATC | Quantification of *Cetobacterium* |

**Supplementary Table S2. The developmental stages of TR in tadpoles upon exposure to TR for 21 days.**

| TR concentrations | 0 μg L^−1^ | 1 μg L^−1^ | 10 μg L^−1^ | 100 μg L^−1^ |
| --- | --- | --- | --- | --- |
| Developmental stage | 57.5^A^ | 56.5^AB^ | 55.5^B^ | 55.5^B^ |

*n* = 6 for each. Developmental stages are represented as the median. Different letters indicate a significant difference based on a one-way analysis of variance ANOVA with LSD test (*p* < 0.05).

**Supplementary Table S3. Dominant bacterial phyla of the gut microbiota in tadpoles at different groups.**

| Phylum | Control (%) | TR (%) |
| --- | --- | --- |
| Firmicutes | 23.72 | 26.49 |
| Bacteroidetes | 25.70 | 21.05 |
| Verrucomicrobia | 17.83 | 17.51 |
| Proteobacteria | 18.42 | 14.24 |
| Fusobacteria | 8.09 | 19.74 |
| Others | 6.24 | 0.97 |

**Supplementary Table S4. Percentage of the core taxa in the gut microbiome between the control and TR-treated groups (10 μg L^−1^) tadpoles.**

| Taxa | Control (%) | TR (%) |
| --- | --- | --- |
| *Fusobacterium* | 6.80 | 17.60 |
| *Akkermansia* | 17.80 | 16.77 |
| *Desulfovibrio* | 14.81 | 10.89 |
| f:Lachnospiraceae | 5.45 | 8.49 |
| *Bacteroides* | 6.58 | 8.38 |
| *Eubacterium* | 4.91 | 5.49 |
| *Cetobacterium* | 1.29 | 2.14 |
| *Romboutsia* | 4.87 | 2.13 |
| *Odoribacter* | 2.74 | 1.84 |
| f:Tannerellaceae | 2.98 | 1.57 |
| *Acetobacterium* | 1.24 | 1.44 |
| *Parabacteroides* | 1.68 | 0.88 |
| Others | 28.85 | 22.38 |


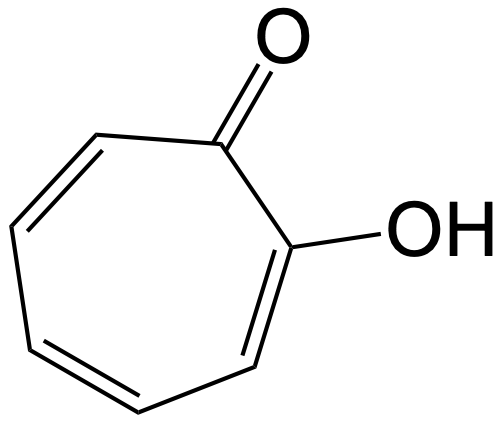


**Fig. S1. The chemical structure of biotoxin tropolone (TR).**


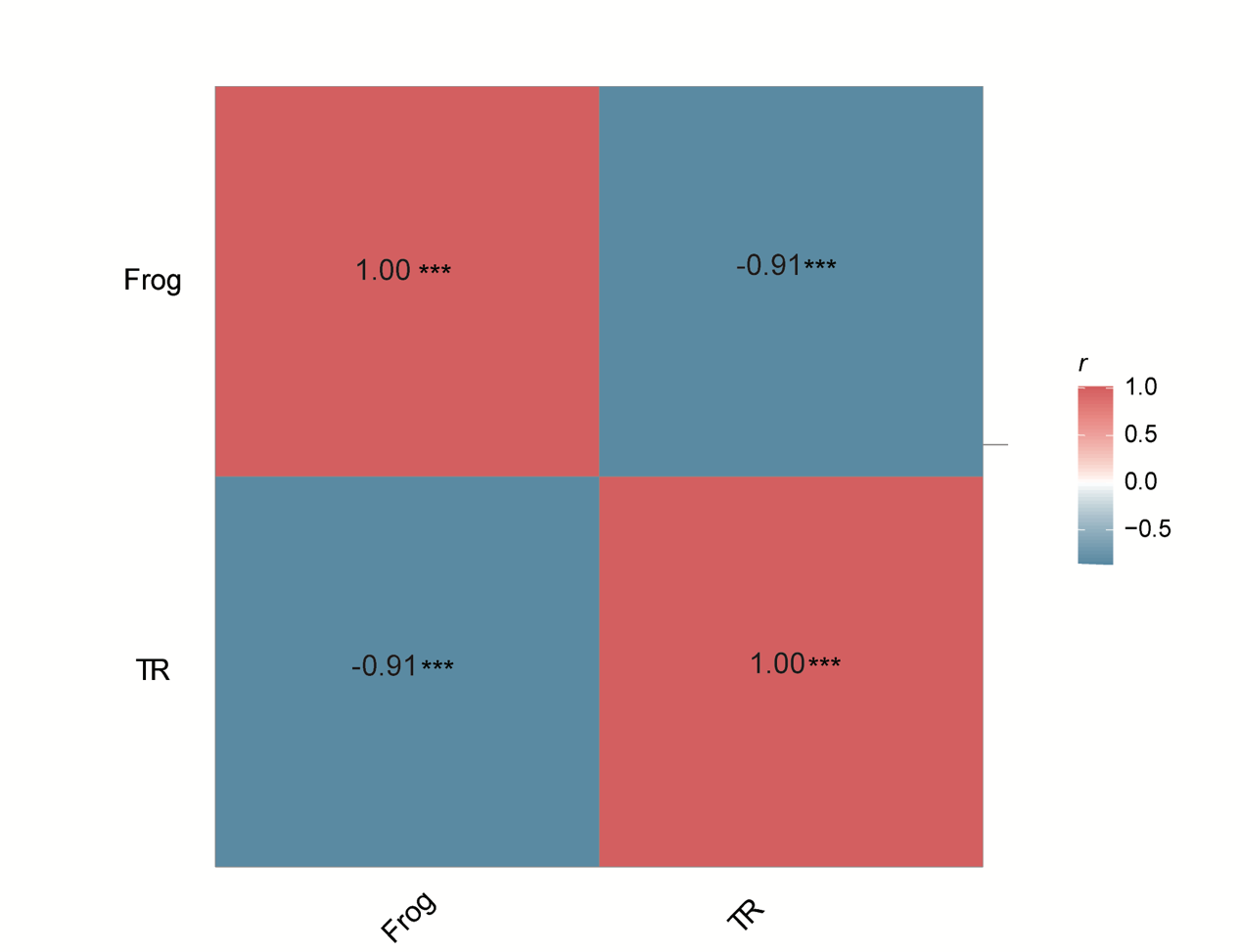


**Fig. S2.** **The density of frogs is negatively correlated with TR in paddy fields.** Heatmap of Pearson’s correlation coefficients. Asterisk (***) indicates a significant correlation at *p* < 0.001.

**
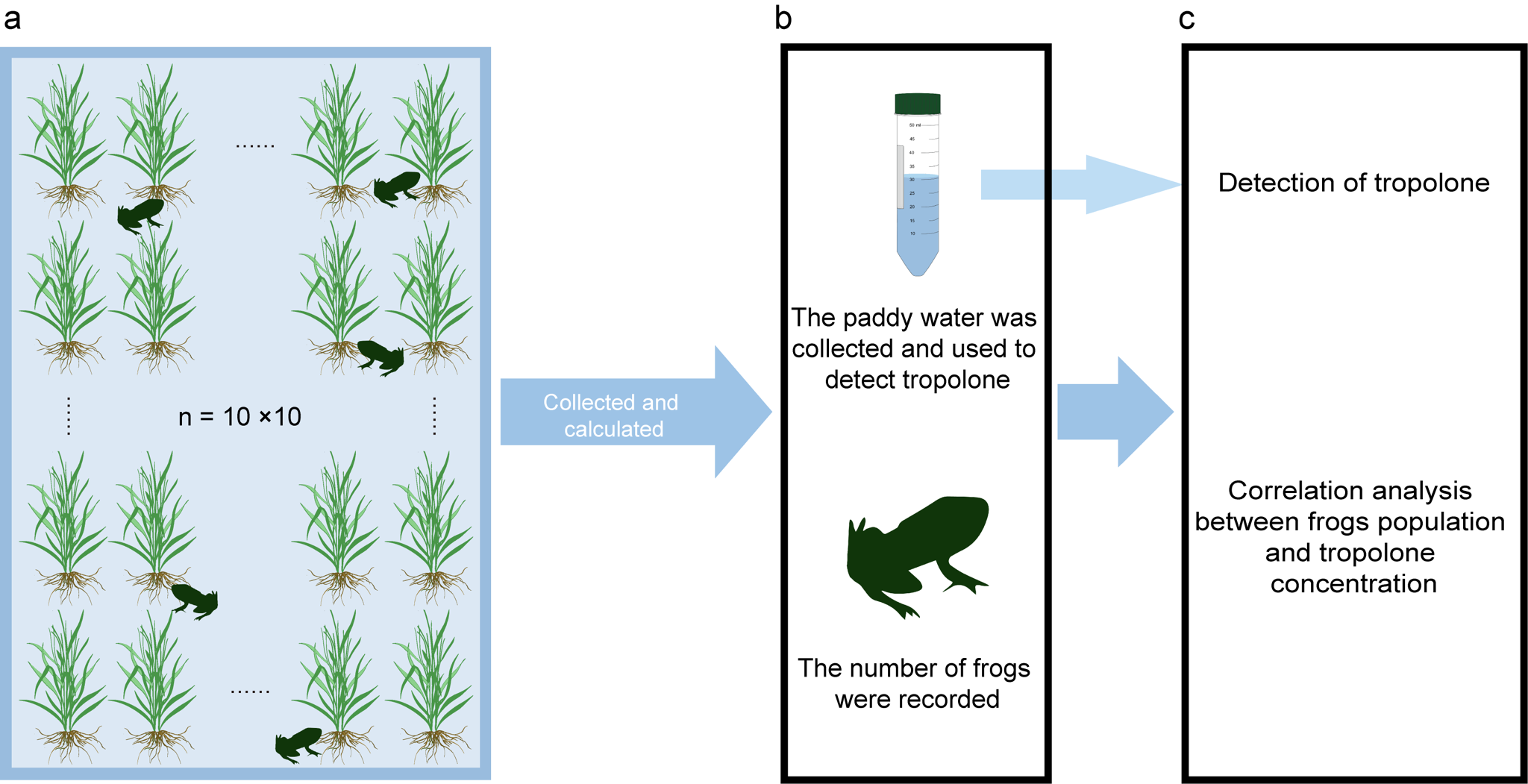
**

**Fig. S3. The flow chart of the field sampling and the simplified workflow of subsequent analysis. a–b,** Field sampling in paddy fields. The chessboard-type sampling was applied to calculate the population of frogs in 100 plants per paddy field. The five-point sampling method was implemented to collect and mix the paddy water. The samples were transported to the Laboratory. **c,** The subsequent experiment and analysis. The correlation analysis between frog populations and TR concentrations.


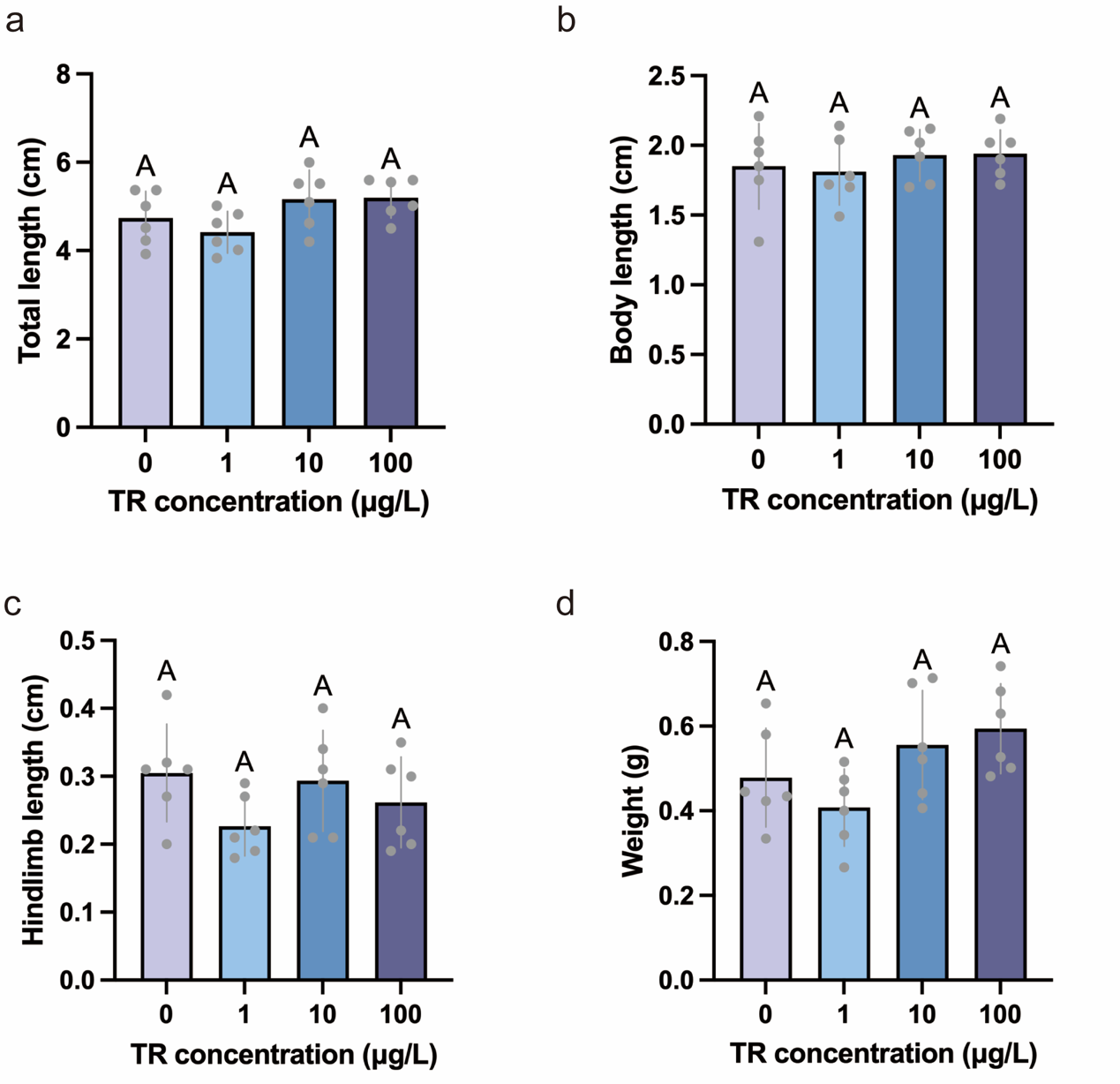


**Fig. S4. Alterations of development in the tadpoles upon exposure to TR at seven days. a–d**, Effects of TR exposure (0, 1, 10, 100 μg L^-1^) on the total length (a), body length (b), hindlimb length (c), and weight (d) of tadpoles. *n* = 6 for replicates. Above all have the different letters with error bars which indicate a significant difference by one-way analysis of variance (ANOVA) with LSD test (*p* < 0.05).


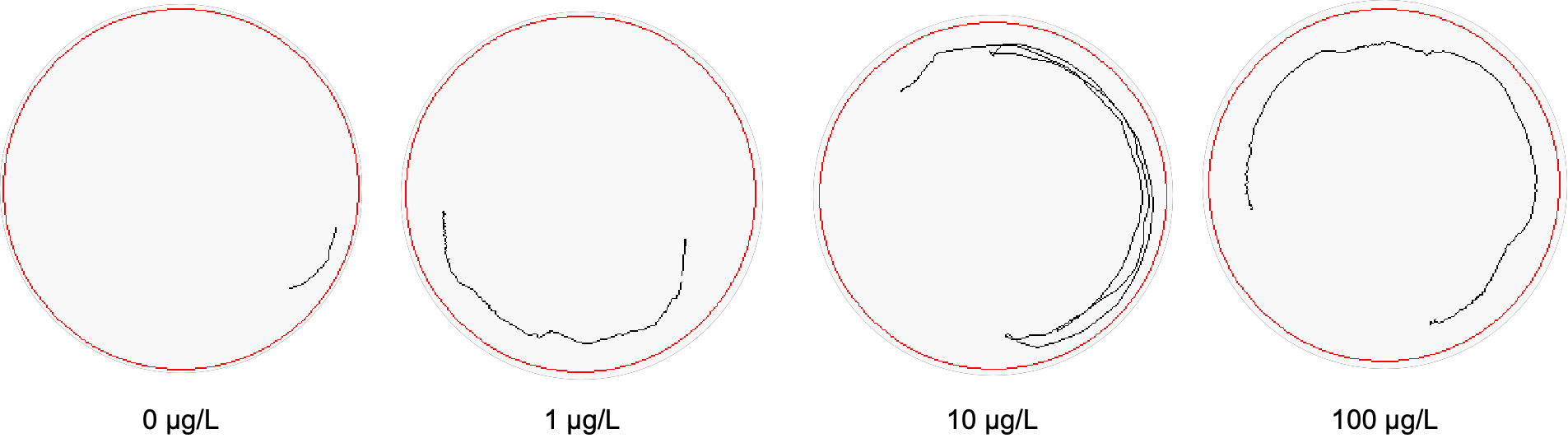


**Fig. S5.** **The representative individual tracking of tadpoles in the control (TR at 0 μg L^−1^) and the TR-exposed groups (TR at 1, 10, 100 μg L^−1^).** The tracking of tadpoles was recorded every 10 seconds.


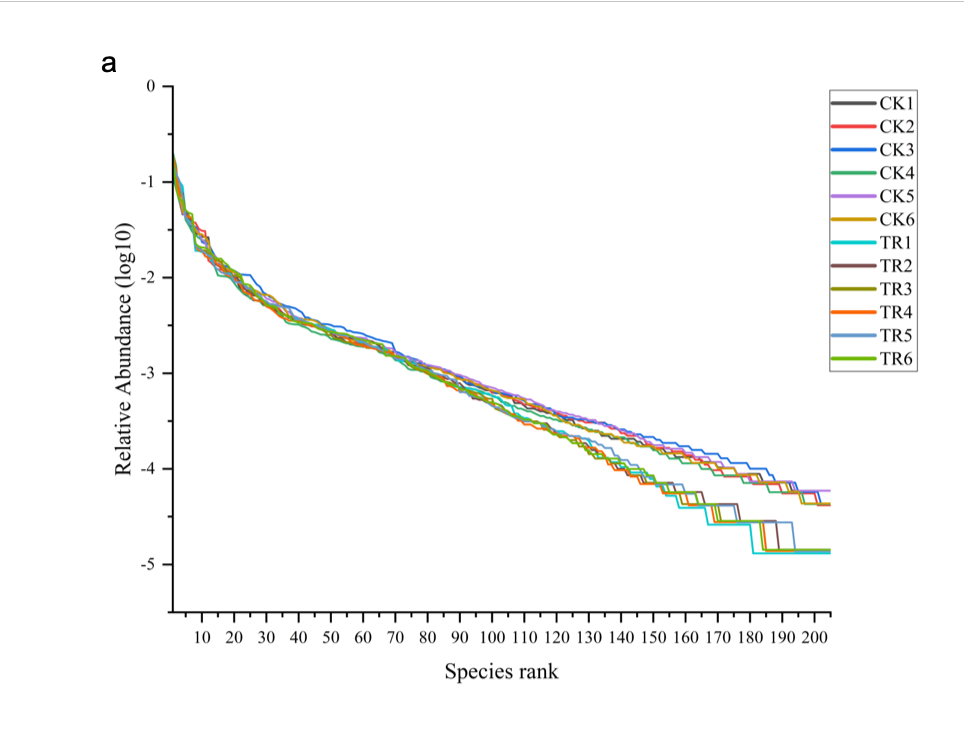

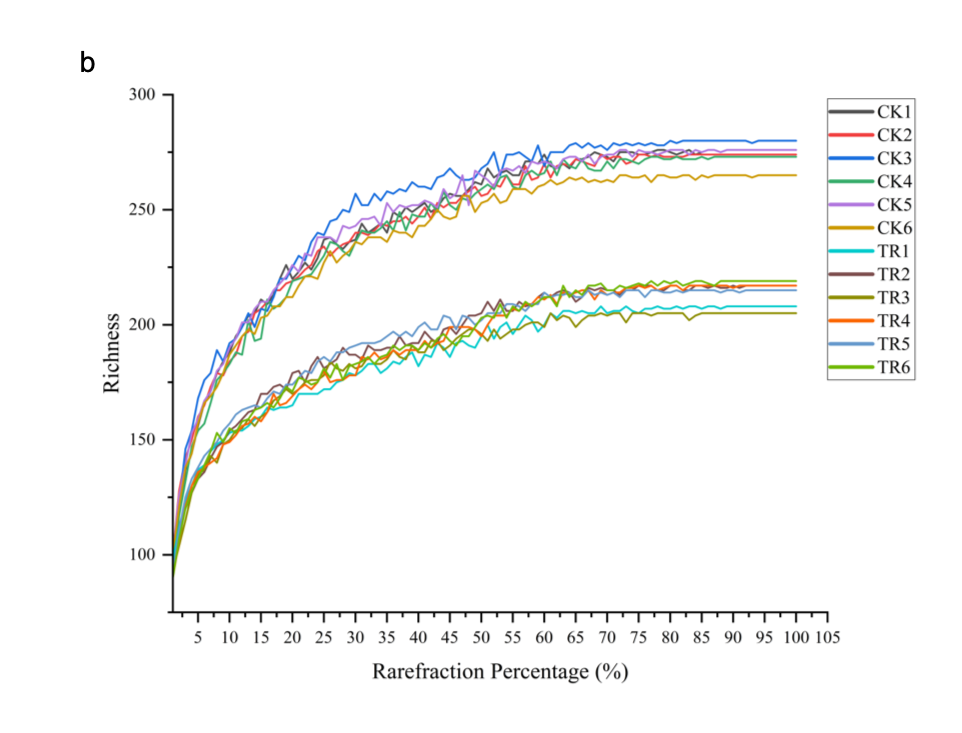


**Fig. S6. Overall structure of the gut microbiota of tadpoles upon exposure to 10 μg L^−1^ concentration of TR. a,** Rank-abundance curve of microbial species in each sample (*n* = 6). **b,** Rarefaction curves of 16S rRNA in samples of tadpoles in the control and TR-exposed groups (*n* = 6).

**
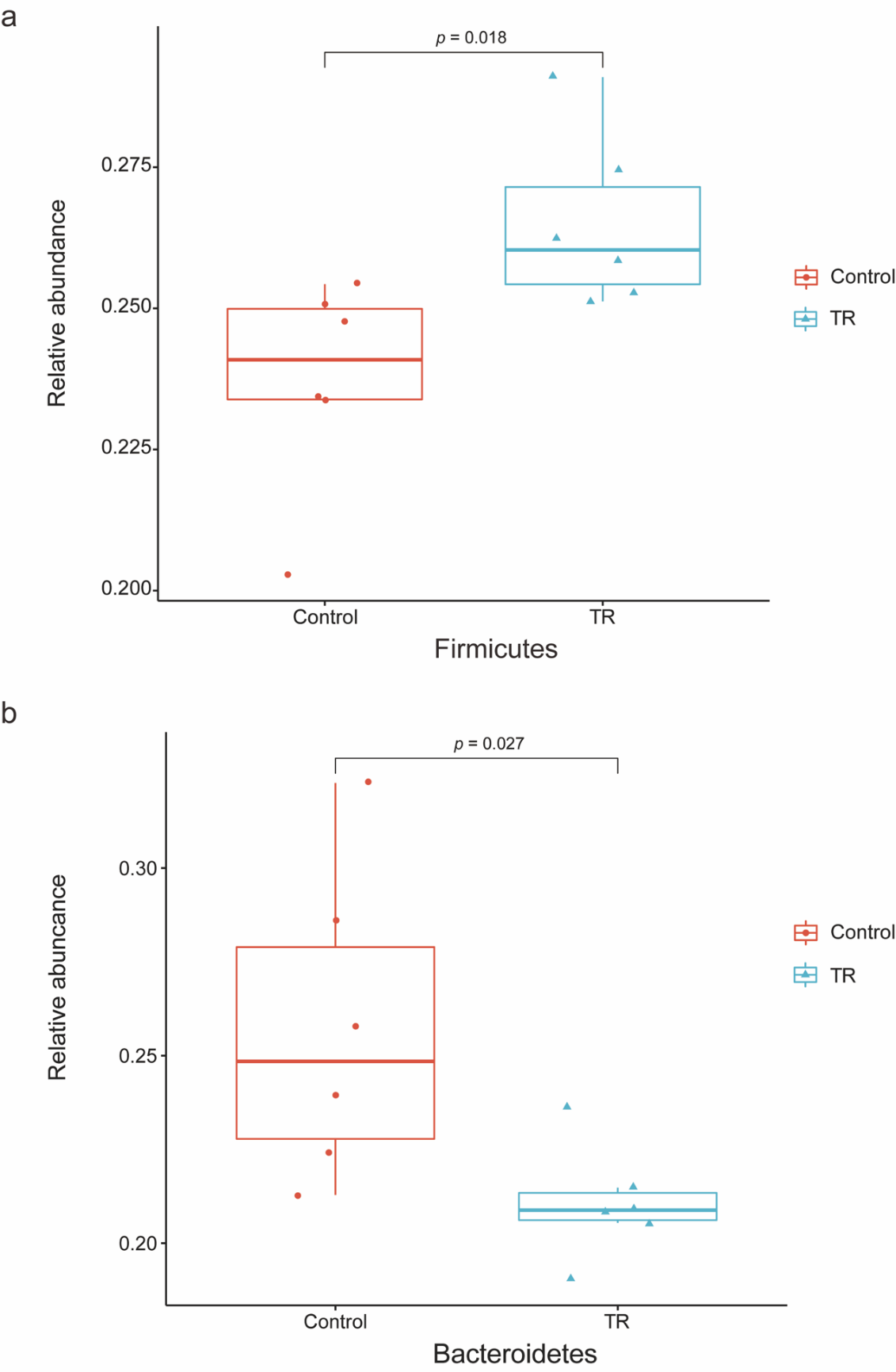
**

**Fig. S7. The boxplot of the dominated phyla of the tadpoles’ gut microbiome in the control (TR at 0 μg L^−1^) and the TR-exposed (10 μg L^−1^) groups. a, b,** The boxplots of phyla Firmicutes (**a**) and Bacteroidetes (**b**). *n* = 6 replicates.

**
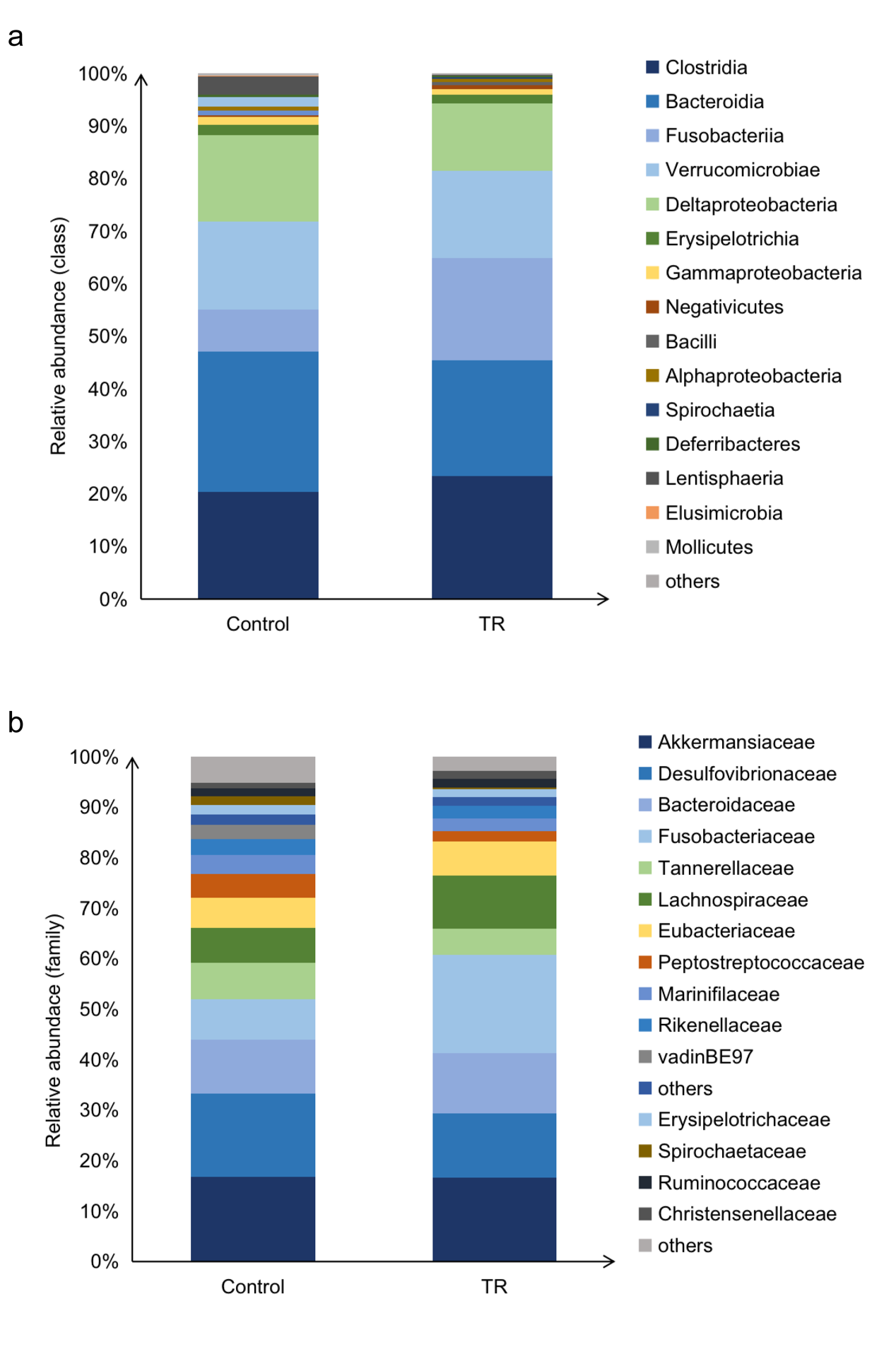
**

**Fig. S8. Bacterial composition of the gut microbiome in tadpoles.** The stacked histograms illustrate the class-level (**a**) and family-level (**b**) bacterial composition of the gut microbiome of tadpoles in the control (TR at 0 μg L^−1^) and the TR-exposed (10 μg L^−1^) groups.

#
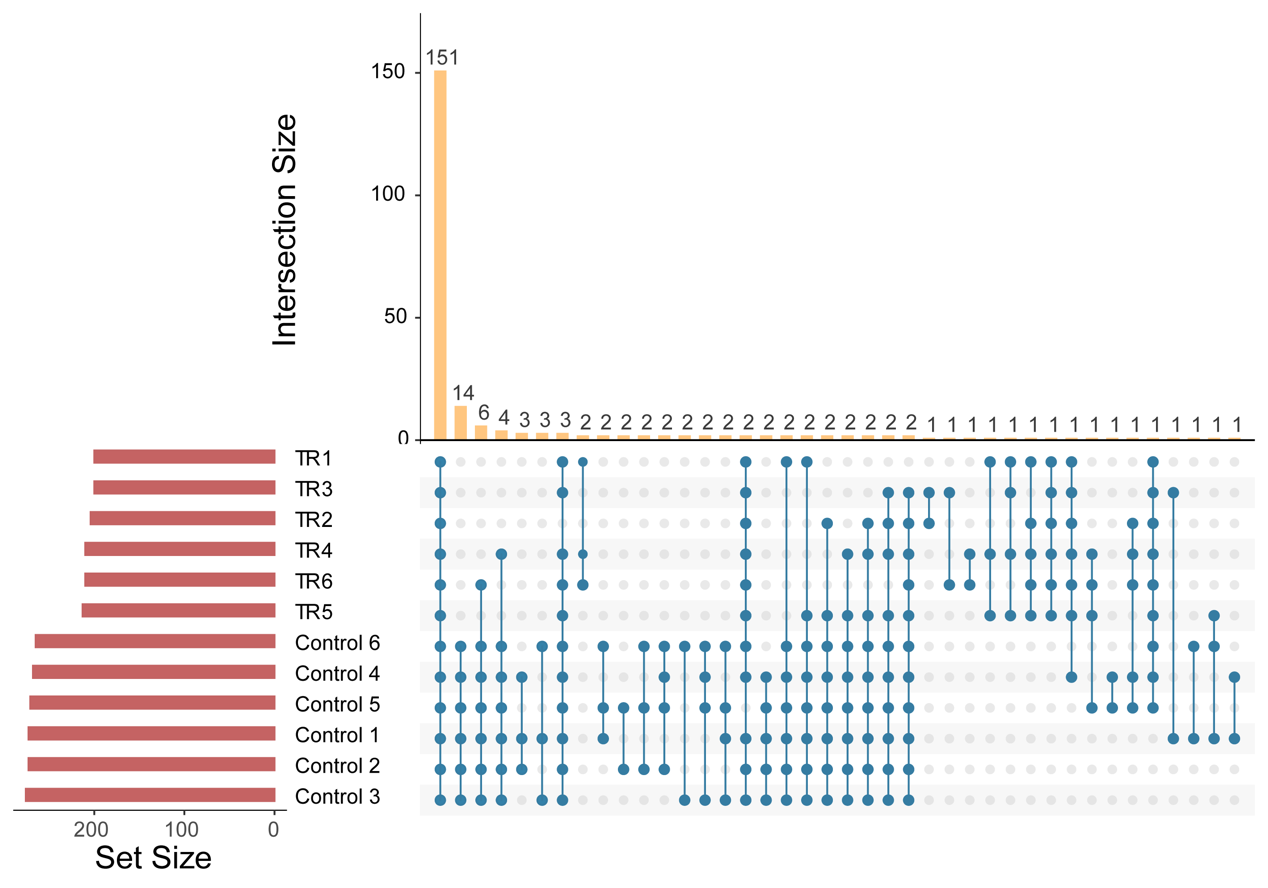


**Fig. S9. UpSet plot of the gut microbiome in the TR-exposed (10 μg L^−1^) tadpoles.** The length of the red bars (bottom left) indicates the total size sets of the OTUs. The blue symbols connected with blue lines represent the intersections between these sets and the number, while the yellow columns indicate the frequency of these intersections. *n* = 6 replicates.

**
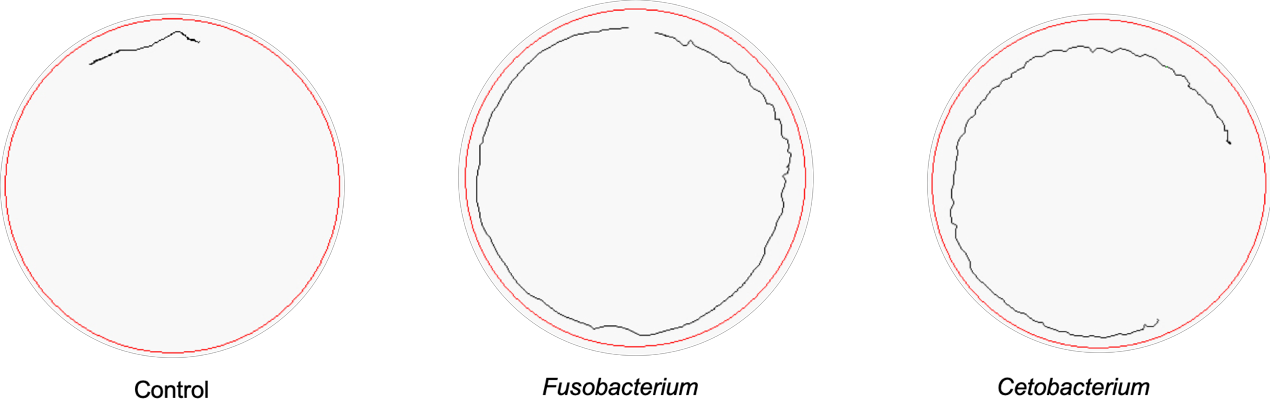
**

**Fig. S10. The representation of individual tracking of tadpoles in the control groups and the transplantation of core taxa bacteria groups.** The tracking of tadpoles was recorded every ten seconds.


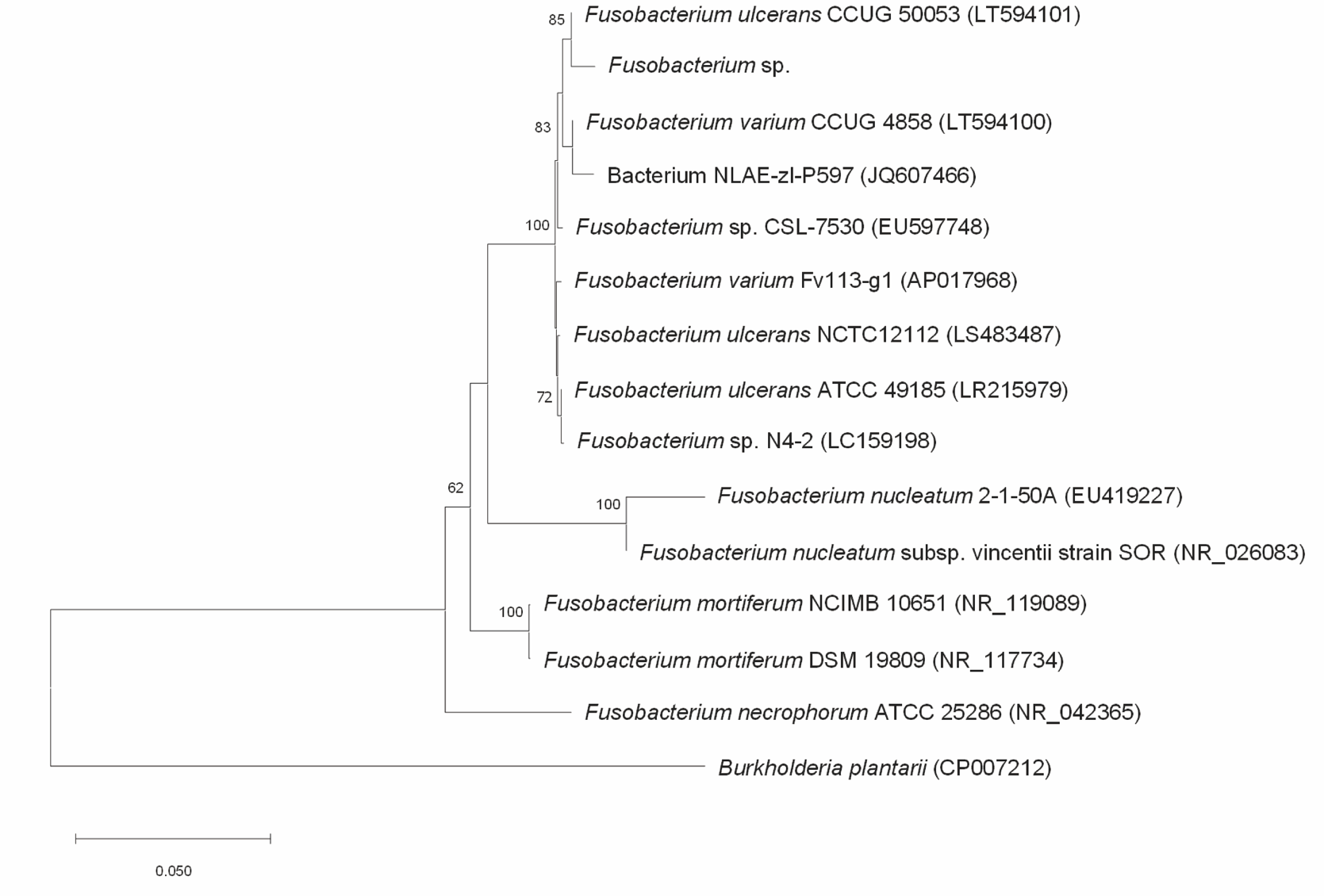


**Fig. S11.** **Phylogenetic analysis of the 16S rRNA gene sequence of *Fusobacterium* sp.** The phylogenetic tree was constructed by the Neighbor-joining method using MEGA, with the lengths of the horizontals proportional to the differences.


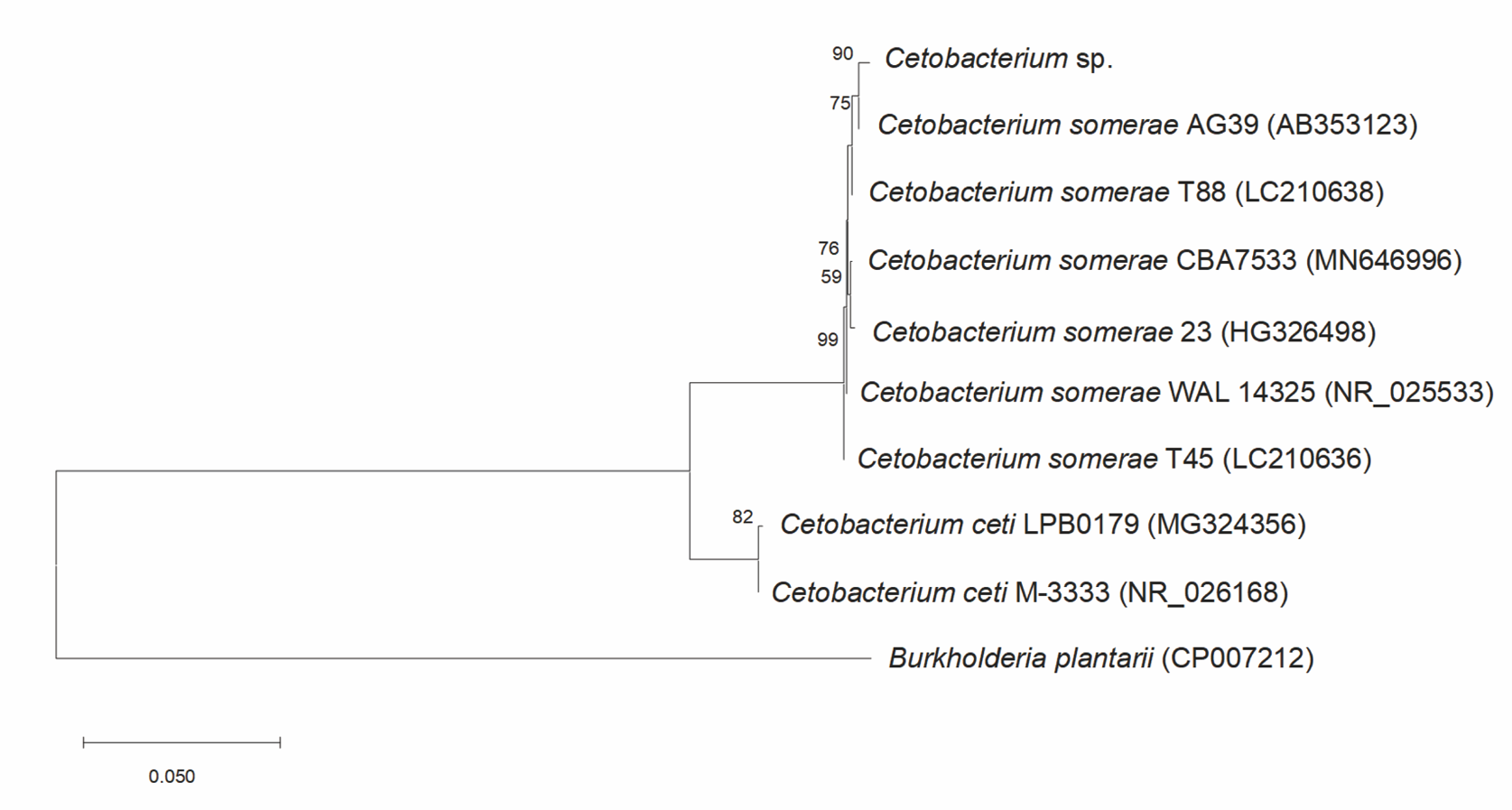


**Fig. S12. Phylogenetic analysis of the 16S rRNA gene sequence of *Cetobacterium* sp.** The phylogenetic tree was constructed by the Neighbor-joining method using MEGA, with the lengths of the horizontals proportional to the differences.


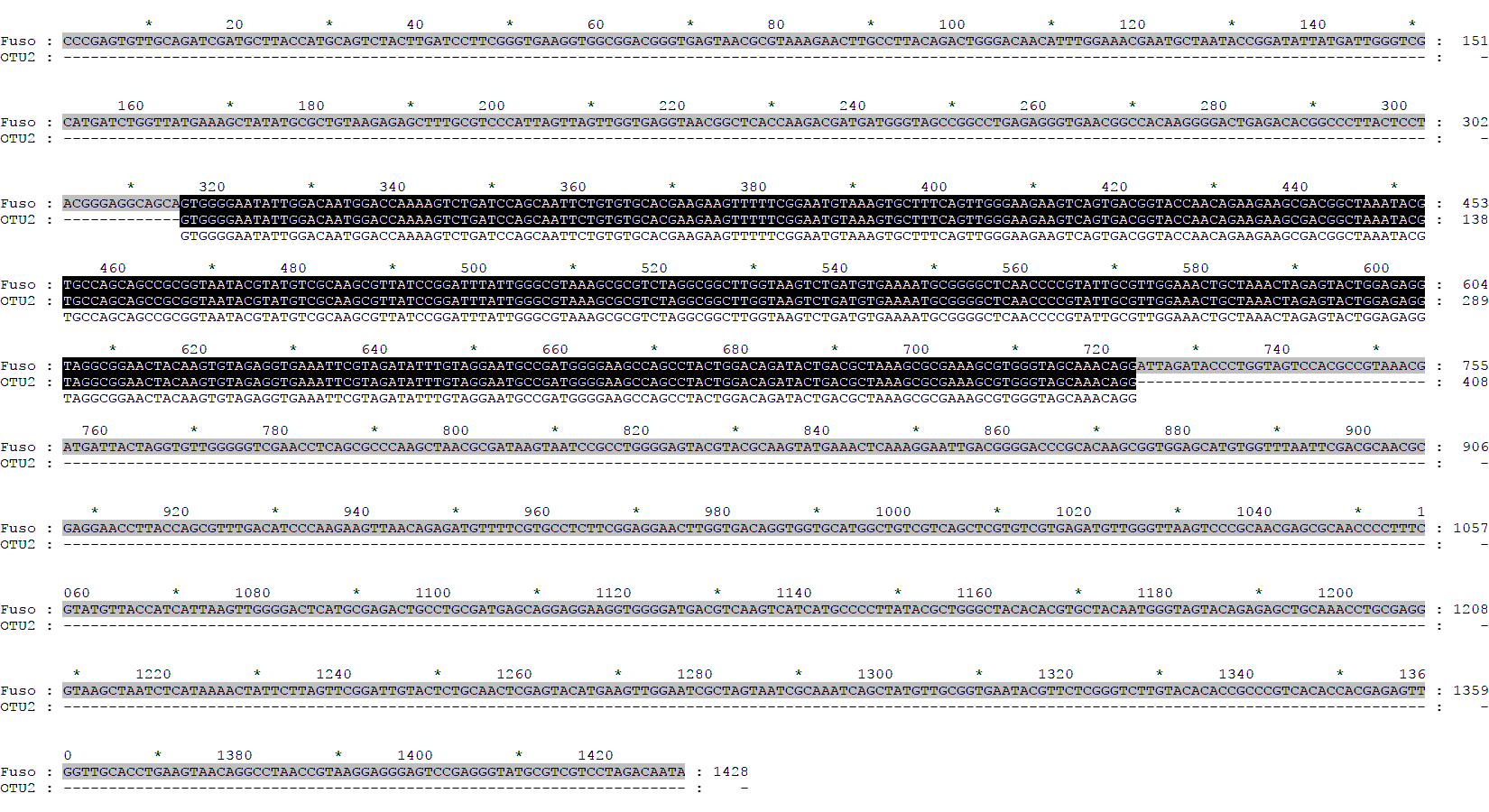


**Fig. S13.** **Sequence alignment of *Fusobacterium* strains.** Fuso indicates the 16S rRNA gene sequence of *Fusobacterium* sp., isolated from the gut of tadpoles. The sequence of OTU2 is obtained from the microbiome profiling.


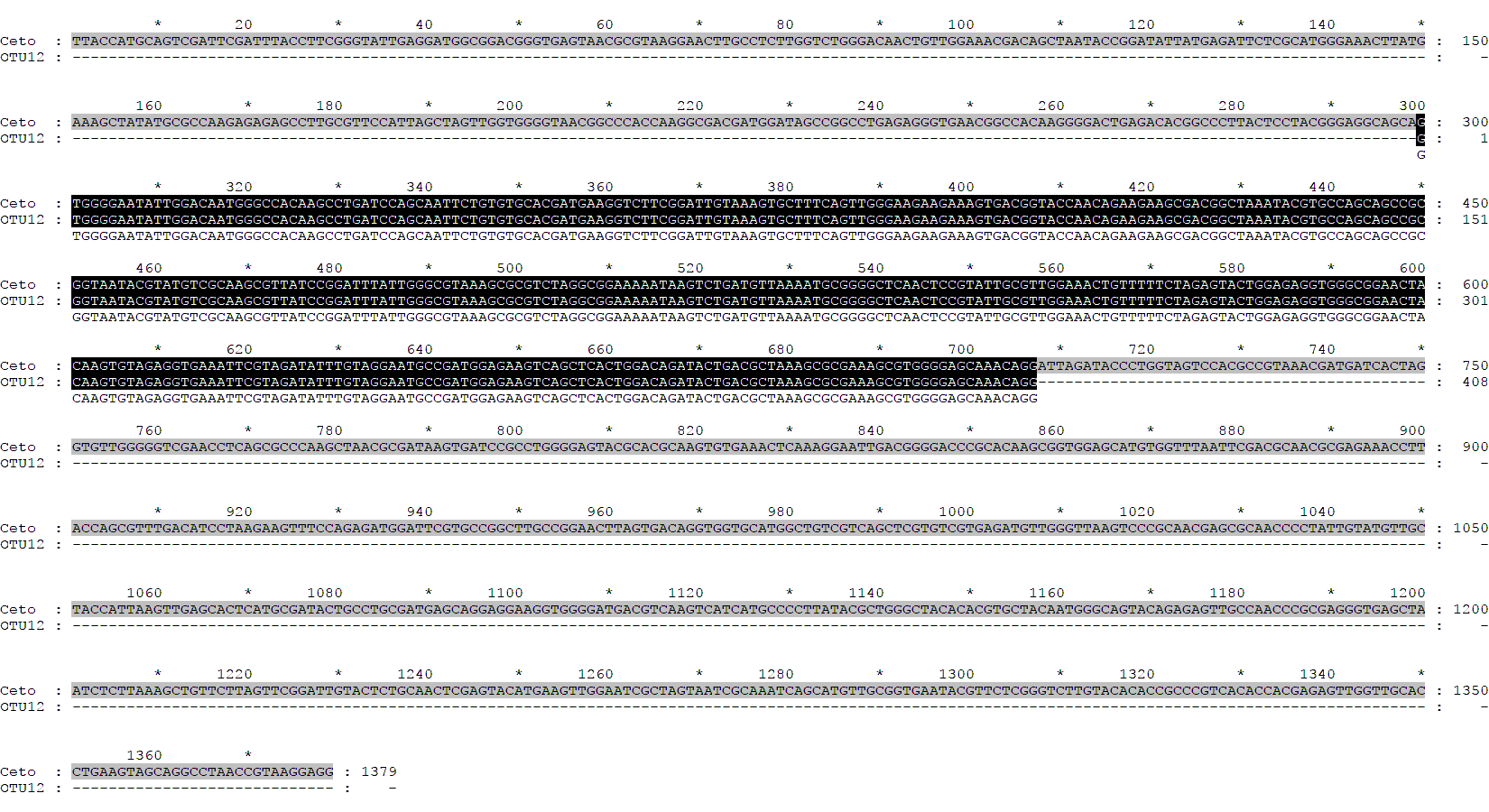


**Fig. S14. Sequence alignment of *Cetobacterium* strains.** Ceto indicates the 16S rRNA gene sequence of *Cetobacterium* sp., isolated from the gut of tadpoles. The sequence of OTU12 is obtained from the microbiome profiling.

**
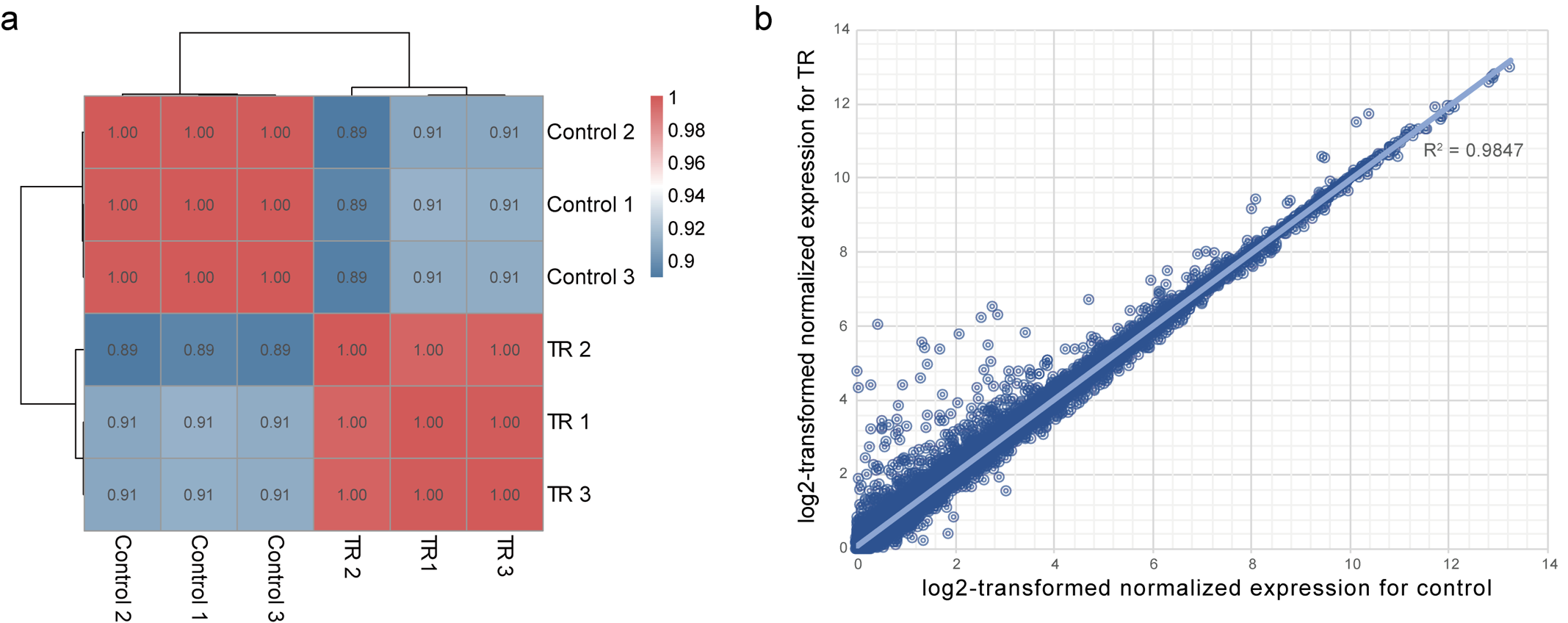
**

**Fig. S15. Reliability testing for the transcriptome analysis of TR-treated (10 μg L^−1^) tadpoles. a,** The colors of plots display the degree between the control and TR exposure groups and the number of the plots indicates the correlation coefficient *r* values. **b,** The light blue line in the plot represents the simple linear regression analysis of the values between the log_2_-transformed normalized gene expressions of the control (control 1, control 2, and control 3) and the TR-exposed (TR 1, TR 2, and TR 3) samples. For each transcriptome pair, Pearson’s correlation coefficients (R^2^ = 0.9847) indicated the data repeatability (*p* < 0.001, Student’s *t*-test, two-tailed).

**
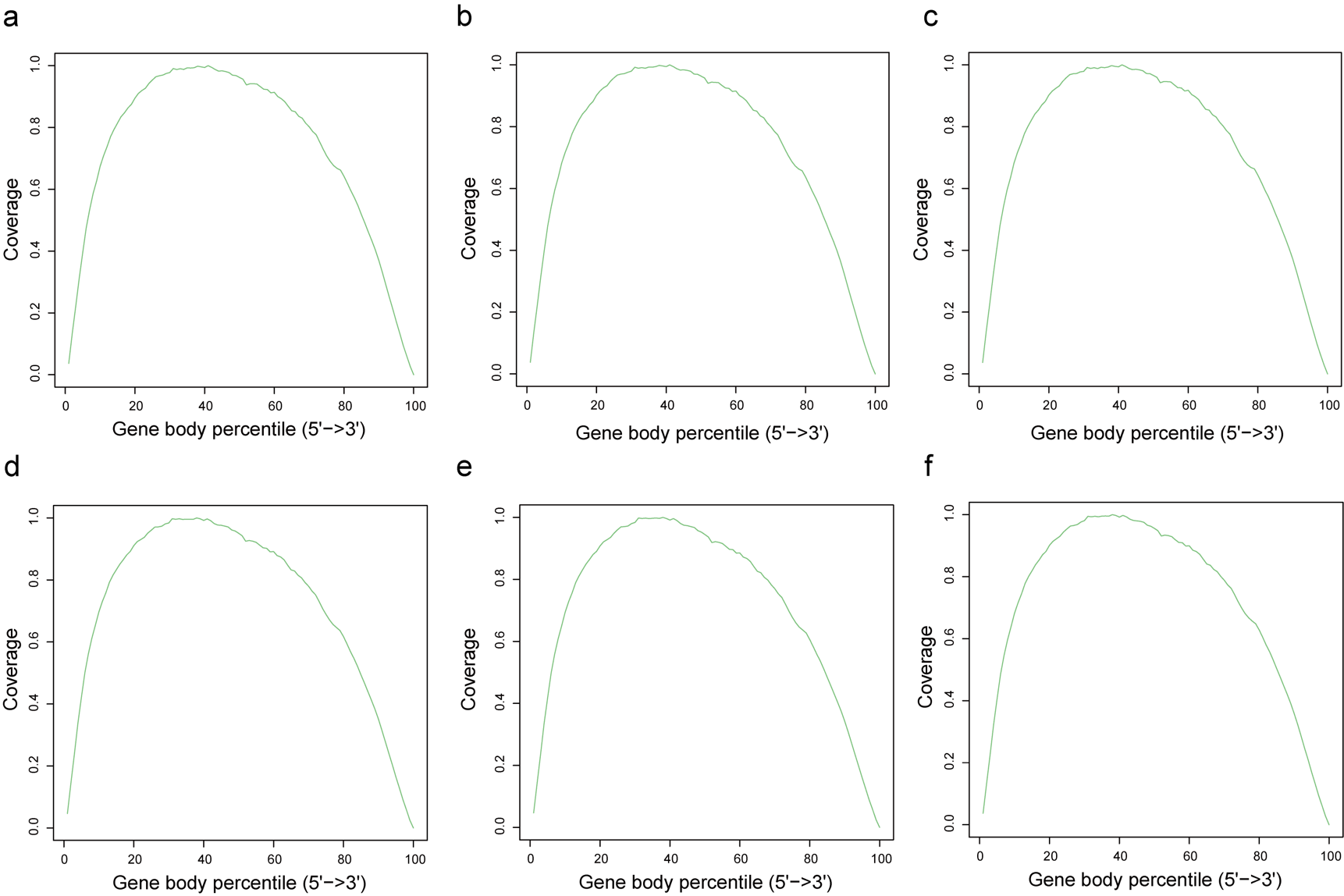
**

**Fig. S16. Gene body coverage profile for each sample.** **a–c,** Gene body coverage profile for control groups. **d–f**, Gene body coverage profile for TR-exposed groups.

**
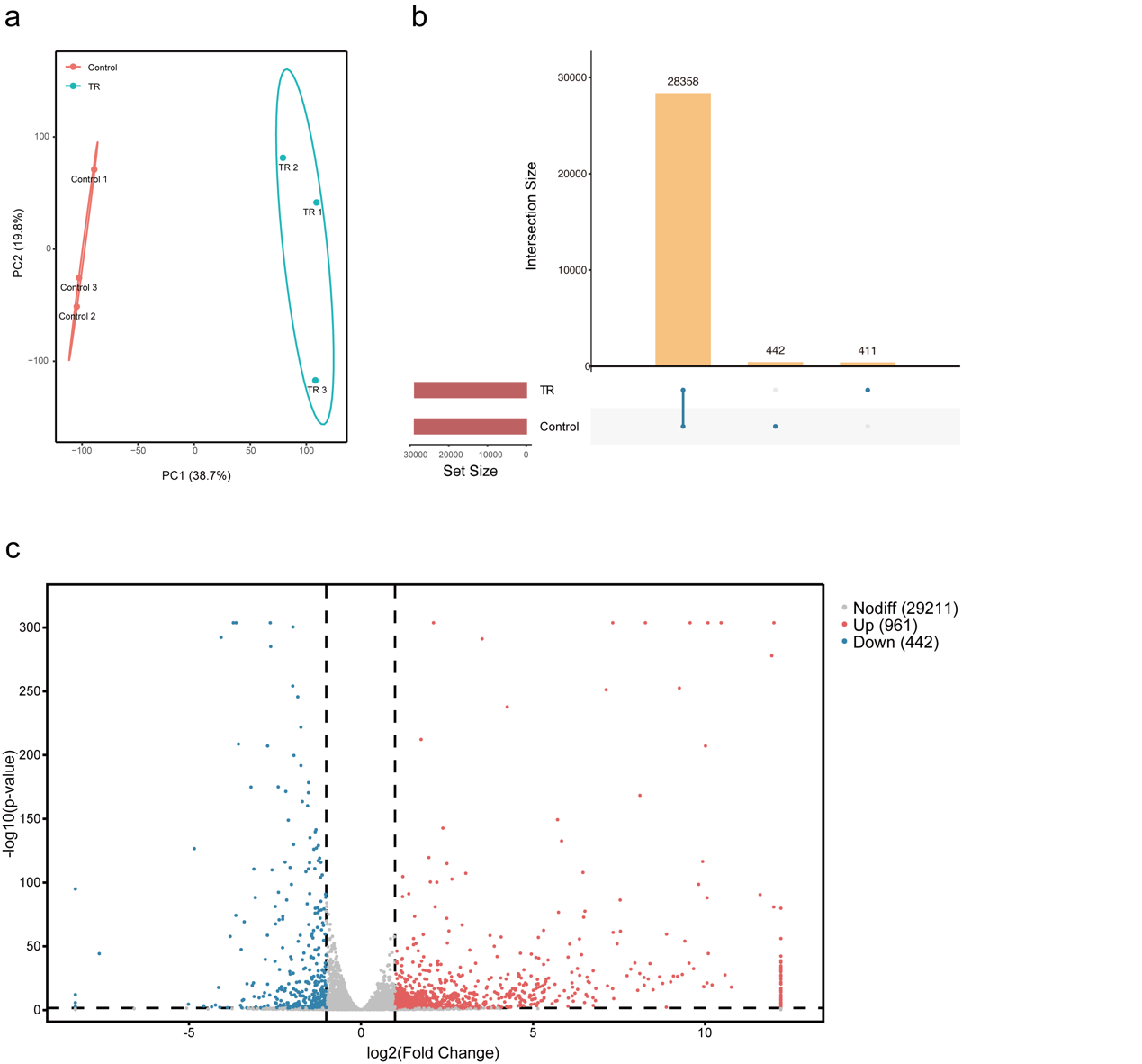
**

**Fig. S17. Analysis of the differentially expressed genes in tadpoles upon exposure to 10 μg L^−1^ concentration of TR. a,** PCA analysis of RNAseq. *n* = 3 replicates. **b,** UpSet plot demonstrates the genes of tadpoles are significantly changed in tadpoles upon exposure to TR. The length of the red bars (bottom left) indicates the total size sets of the differentially expressed genes. The blue symbols connected with blue lines represent the intersections between the sets and the number, while the yellow columns indicate the frequency of these intersections. *n* = 3 replicates. **c,** Transcriptome pattern in tadpoles upon exposure to TR. The red dots represent the up-regulated genes, while down-regulated genes are shown in blue. The grey dots in the center are not significantly altered.

**
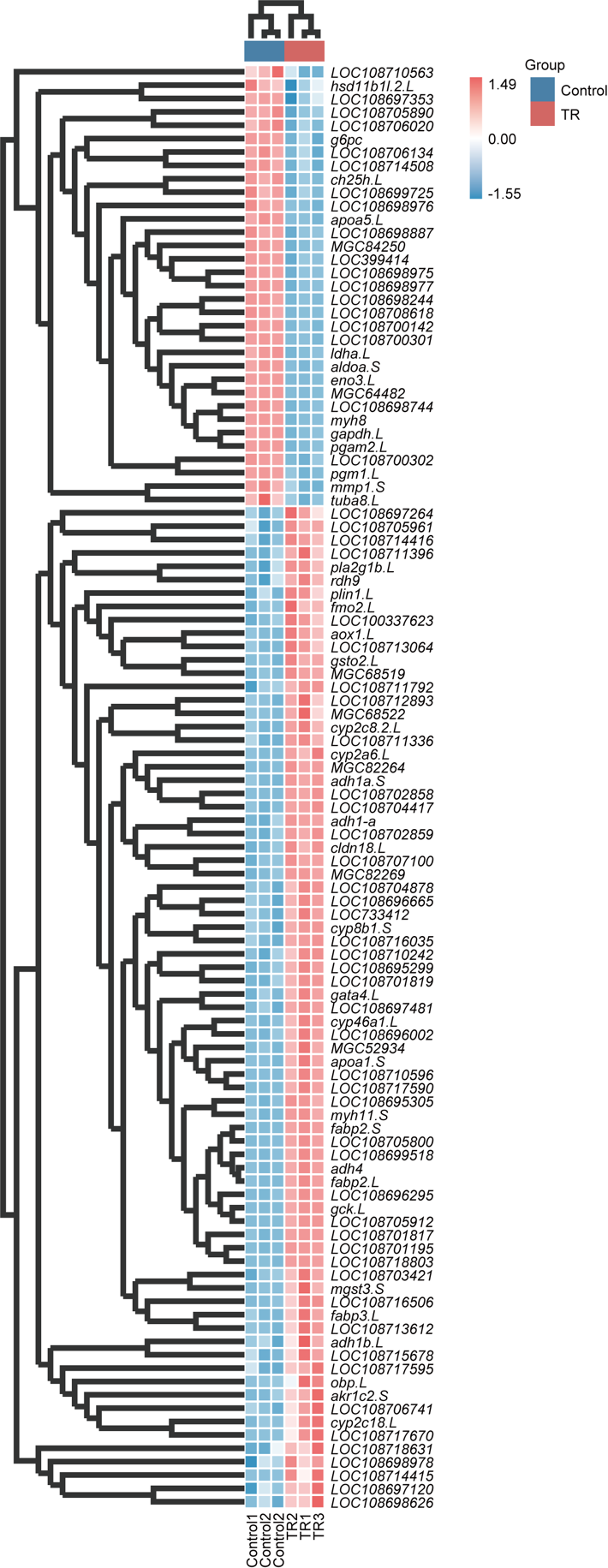
**

**Fig. S18. The cluster heatmap of the difference expression genes of the top 10 KEGG pathways in tadpoles upon exposure to TR (10 μg L^−1^).** Hierarchical clustering of 108 genes that are differentially expressed in the RNAseq. A fitted generalized linear model products heatmap that shows fold changes derived from log_2_-transformed fold changes in the treatment groups compared with the control groups.

**
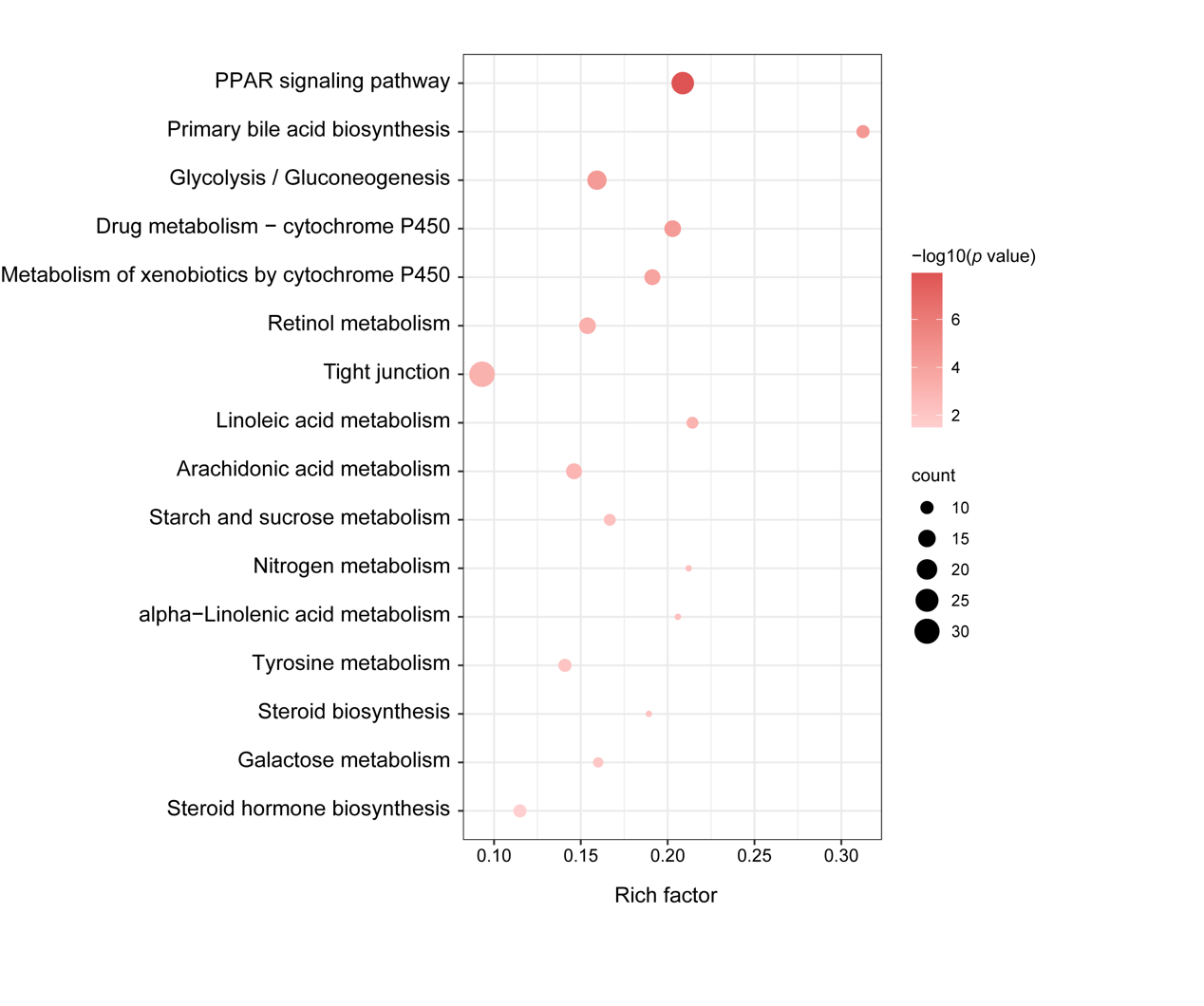
**

**Fig. S19. The scatter plot of the top 16 significantly enriched KEGG pathways in tadpoles upon exposure to 10 μg L^−1^ concentration of TR.** The rich factor represents the radio of the DEGs number to the total gene number in a certain pathway. The size and color of dots indicate the number of DEGs mapped to the related pathways and the range of the *p* value of KEGG pathways enrichments by -log_10_-transformed, respectively.

**
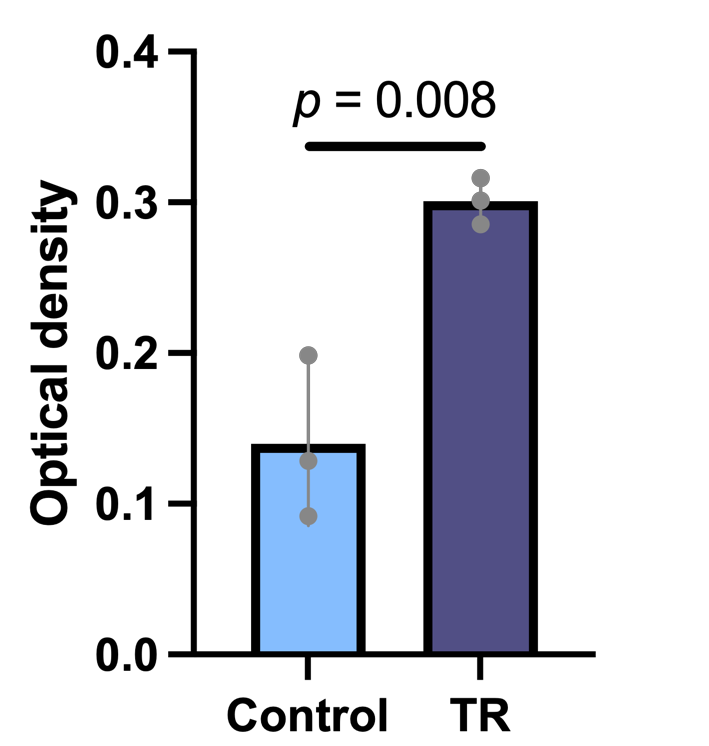
**

**Fig. S20. Determination of dopamine in the tadpoles upon exposure to 10 μg L^−1^ concentration of TR.** The concentration of dopamine was detected by optical density at 450 nm.
